# One year results of a randomized controlled clinical study evaluating the effects of non-surgical periodontal therapy of chronic periodontitis in conjunction with three or seven days systemic administration of amoxicillin/metronidazole

**DOI:** 10.1371/journal.pone.0179592

**Published:** 2017-06-29

**Authors:** Raluca Cosgarea, Christian Heumann, Raluca Juncar, Roxana Tristiu, Liana Lascu, Giovanni E. Salvi, Nicole B. Arweiler, Anton Sculean

**Affiliations:** 1 Clinic of Periodontology, Philipps University, Marburg, Germany; 2 Clinic of Prosthodontics, University of Medicine and Pharmacy Iuliu Hatieganu, Cluj-Napoca, Romania; 3 Department for Statistics, Ludwig-Maximilians University, Munich, Germany; 4 Department of Periodontology, University of Bern, Bern, Switzerland; Centre for Dental, Oral, and Maxillofacial Medicine, GERMANY

## Abstract

**Background:**

To evaluate the clinical outcomes 12 months after systemic administration of amoxicillin (AMX) and metronidazole (MET) adjunctive to subgingival debridement (SD) in patients with severe chronic periodontitis (sChP).

**Material and methods:**

102 patients with sChP were treated randomly as follows: SD within 2 consecutive days and placebo for 7 days (group A), SD+AMX+MET (both 500mg x3 times daily TID) for 3 days (group B), SD+AMX+MET (both 500mg x 3 TID) for 7 days (group C). At baseline, at 3-, 6-, and 12-months post-treatment probing pocket depth (PD), clinical attachment level (CAL), furcation involvement, bleeding on probing (BOP), full-mouth plaque score (FMPS) were determined. The reduction in the number of sites with PD≥6mm was defined as main outcome variable.

**Results:**

75 patients completed the study. At 12 months, all three treatment groups showed statistically significant improvements (p<0.001) of mean PD, CAL, BOP and number of sites with PD≥6mm compared to baseline. Mean residual PD were statistically significantly lower and CAL gain statistically significantly greater in the two antibiotic groups as compared to placebo. While PD reductions (p = 0.012) and CAL gain (p = 0.017) were statistically significantly higher in group C compared to group A, only the 3-day AB group showed statistically significantly fewer sites with PD≥6mm at 12 m (p = 0.003). The reduction in the number of sites with PD≥6 mm (primary outcome) showed no statistical significant differences between the 3 treatment groups. However, in both antibiotic groups significantly more patients compared to the placebo group reached a low risk for disease progression at 12 months (≤4 sites with PD≥5mm).

**Conclusion:**

At 12 months, both adjunctive antibiotic protocols resulted in statistically significantly greater clinical improvements compared to placebo.

## Introduction

It is widely accepted that chronic periodontitis (CP) is a plaque-induced inflammatory disease, that affects tooth-supporting structures. If left untreated, the progressive destruction of these tissues may ultimately result in tooth-loss [1]. Therefore, the main focus of periodontal therapy is the removal of bacterial deposits from the root surfaces through mechanical debridement [i.e. scaling and root planning (-SRP)] aiming at resolving the inflammation, arresting disease progression and re-establishing periodontal health. Several authors provided evidence that persistence of *Aggregatibacter actinomycetemcomitans (A*.*actinomycetemcomitans)*, *Porphyromonas gingivalis (P*. *gingivalis)* and some other bacterial species are associated with the progression of tissue destruction [2–4] while better clinical outcomes were obtained when these bacteria were undetectable [5–7]. Therefore, reduction or even complete eradication of the microbial load seems to be a necessity for obtaining long-term clinical stability [8, 9].

In order to accomplish these goals, non-surgical periodontal therapy is frequently combined with adjunctive antibacterial agents i.e. antibiotics and antiseptics [3, 10–13]. A large number of studies provided evidence indicating that the adjunctive use of Amoxicillin (AMX) and Metronidazole (MET) to SRP had positive effects on the suppression of *A*. *actinomycetemcomitans* and other periodontopathogenic bacteria from periodontally compromised sites [14–18]. Statistically and clinically significantly better clinical outcomes in terms of probing pocket depth (PD) reduction and clinical attachment level (CAL) gain were observed when the combination of AMX + MET was administered adjunctively to SRP compared to treatment with SRP alone [10, 11, 19–29]. Additionally, this adjunctive antibiotic treatment regimen yielded a statistically significant reduction of periodopathogenic bacteria and of inflammatory cytokines compared to SRP alone [19–31].

Even though a large variety of studies focused on the clinical, microbiological and inflammatory effects of the adjunctive use of AMX and MET to SRP, no consensus exists regarding the optimal dosage and duration of these antibiotics. While some authors have investigated the adjunctive administration of 375 mg AMX and 250/500 mg MET 3 TID for 7–8 d to SRP [19–21, 32, 33], others have evaluated the effect AMX and MET in a dosage of 500 mg 3 TID for 7 d [18, 22, 29], or 500 mg AMX and 400/250 mg MET 3 TID for 10 to 14 d [17, 23–27, 34]. Since several side effects such as change of taste, headache, diarrhoea, nausea/vomiting, hypersensitivity, renal and liver toxicity, etc., have been reported following the intake of antibiotics over a longer period (e.g. up to 7 days and longer) [19, 35, 36], it appears important that antibiotics are taken in a minimum bactericidal concentration for a minimum duration [37]. It has been suggested that such concepts may maximize the positive effects of antibiotics by limiting the occurrence of the mentioned side effects. Moreover, the use of a high antibiotic concentration for a short-time, may decrease the possibility to develop microbial antibiotic resistance. Additionally, long-time antibiotic protocols may bare a higher risk for a patient to become non-compliant and patient compliance with unsupervised intake of prescribed medication is important for therapy success [38, 39].

Bacterial resistance to antibiotics has been a recognized problem almost from the beginnings of the antibiotic era showing a continuous global increase [40–43]; however, only in the last 20 years, emergence of dangerous, resistant strains with an impact on the success of antimicrobial therapy of life-threatening infections was regularly registered [44, 45]. Antibiotic-resistant infections have a tremendous negative impact on public health and economy: i.e. in the United States, yearly, 2 million people are infected with antibiotic-resistant bacteria and 23 000 die of these infections [46]; furthermore, additional care costs are estimated at 1.6 billion € and 2.5 million € additional hospital days in Europe [45, 47] and 20 billion $ and 8 million $ additional hospital days in US [45, 46, 48]. New resistant strains or resistant factors within bacterial populations can appear especially when low drug doses are prescribed, resulting in poor drug compliance or incomplete penetration of the drug to all tissues [49–51]. Nonetheless, studies investigating antimicrobial resistances observed a decline in resistance frequencies after antibiotic removal [52–54]. Additionally, only a transient increase in the percentage of resistant subgingival species was observed when the antibiotic administration increased, at 90 days after antibiotic therapy the percentage of antibiotic-resistant strains having returned to baseline values [53].

Considering the deleterious effects of prolonged courses of antibiotic therapy (e.g. adverse infections, poor patient compliance, evolution and dissemination of antibiotic resistant organisms, additional high costs and hospitalisation), scientists/doctors in several areas in general medicine tried to optimize the duration of the therapy emphasising the optimal antibiotic combination [55], the implementation of shorter, high-dosage treatment regimes [55–63].

In periodontal therapy, controlled clinical studies evaluating the potential efficacy of a short-term administration of AMX and MET compared to the standard protocol (e.g. use for at least 7 days) are absent. We recently evaluated the clinical outcomes of a 3-day regimen of AMX and MET adjunctive to SRP in severe chronic periodontitis at 6 months showing that both 3 and 7 days adjunctive administration of these antibiotics led to statistically significant better clinical improvements compared to SRP alone at 6 months [11]. However, at present, it is unknown to what extent the results obtained with the short-term administration of AMX and MET remain stable over a longer period of time (e.g. up to one year).

The aim of the present study was therefore to evaluate the clinical outcomes obtained at 1 year following non-surgical periodontal therapy (performed within 2 consecutive days) in conjunction with systemic administration of AMX and MET for 3 or 7 days in patients with severe chronic periodontitis (sCP).

## Material and methods

This study was designed as a randomized, placebo controlled, double-masked clinical trial testing the hypothesis that “the systemic use of AMX and MET administered for 3 or 7 days as adjunct to SD leads to superior clinical results compared with SD alone” [11].

The power of the study was calculated considering a difference of at least 5 sites with a PD ≥ 6 mm [26, 27, 64] and a standard deviation of 6 sites [19, 27] between both AB and placebo groups, respectively. A study power of 92% for a statistical significance level of 0.05 was reached for 30 subjects per treatment group; however, taking into account a possible attrition of 13% we recruited 34 patients per treatment group.

Approval of the Ethical Committee of the Faculty of Medicine and Pharmacy of Cluj-Napoca (Application #514/09.01.2012, approved at 09.01.2012) was obtained for the study protocol, which was conducted according to the Declaration of Helsinki (1964, revision 2008). The study was registered in the ISRCTN trial registry (study ID ISRCTN17605083). Since patient recruitment began in January 2012, and at that time trial registration wasn`t mandatory for many journals, the trial was registered with delay after enrolment of participants has started. The authors confirm that all ongoing and related trials for this drug/intervention are registered.

### Subjects

The clinical protocol was presented in detail previously [11]. Briefly, 102 subjects seeking dental treatment at the Dental University Clinic (University “Iuliu Hatieganu”, Cluj-Napoca) were included and treated as follows: patient recruitment lasted between 10.01.2012–01.03.2014, subgingival debridement was performed between 20.01.2012–30.06.2014 and the follow-up period was between 03.02.2012 (2 weeks post debridement)– 05.06.2015. Following inclusion criteria were considered: age over 30 years, patients had to present at least 12 natural teeth in the oral cavity, with clinical (at least one site/quadrant with PD ≥6mm) and radiographic signs of generalized severe chronic periodontitis [65], good level of oral hygiene (Full-mouth plaque scores ≤ 25% prior SD) [66], systemically healthy with no history of diseases that may influence the severity/progression of periodontitis (e.g. Down Syndrome, HIV, Diabetes Mellitus type 1 and 2), no post-iradiation in the head/neck area, no infectious/heart diseases that need prophylactic administration of antibiotics before dental treatments, absence of liver diseases. Subjects smoking at least 10 cigarettes per day for the last five years were defined as smokers [67]. Patients that had non-surgical periodontal therapy within the previous 12 months, had systemic or local antibiotic therapy within the preceding 3 months, under medication that may have interacted with AMX or MET (e.g., coumarin derivatives, containing alcohol derivatives, 5-fluor-uracyl/ disulfiram derivatives, amprenavir oral solutions, lopinavir/ritonavir oral solution) or under medication with a possible influence on the periodontium (Ciclosporin A, compounds of Phenytoin, calcium channel blockers), or pregnant/lactant patients were excluded from the study. All eligible subjects were thoroughly informed of the aim, risks and benefits of their participation to the study as well as the treatment protocol, and informed written consent to participate in the study was obtained from all participants prior study commencement.

### Clinical protocol

**Fig 1:**
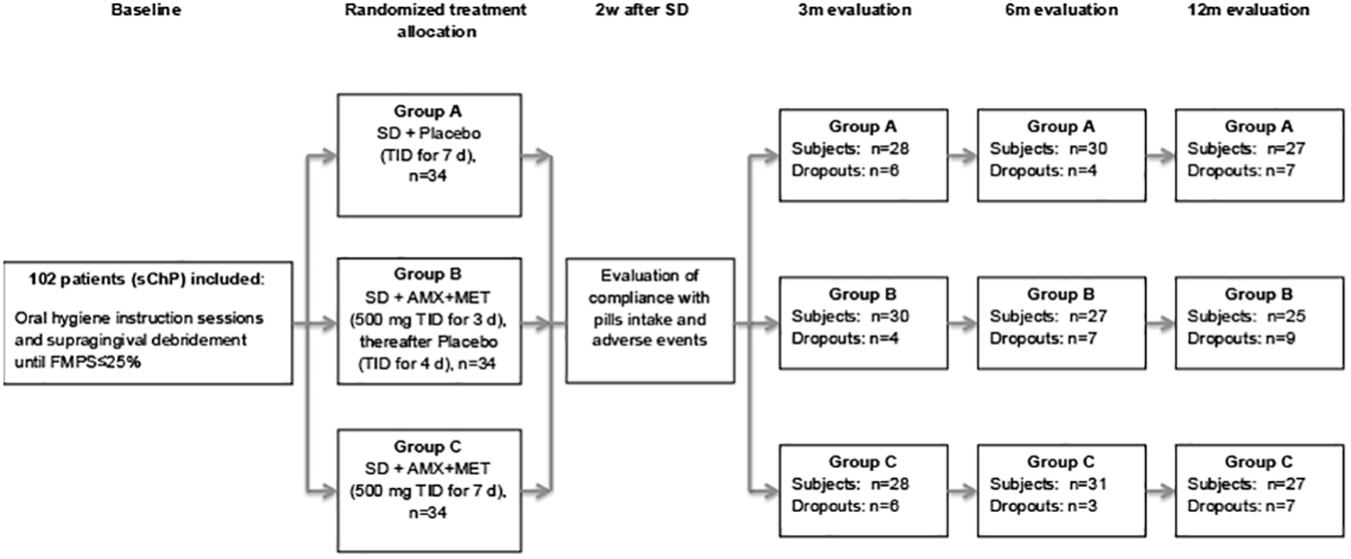
Flowchart of the study. FMPS = full-mouth plaque score; SD = subgingival debridement; ITT = intention to treat analyses; PP = per protocol analyses; m = months.

According to a computer generated list, all included subjects were allocated to one of the three treatment groups: control group A receiving SD + placebo [placebo thrice a day (TID) for 7 days; group B treated with SD and adjunctive systemic AMX+MET (both antibiotics 500 mg TID for 3 days, placebo TID for the rest 4 days; and group C treated with SD and systemic AMX+MET (both antibiotics 500 mg TID for 7 days (Fig 1). Allocation concealment was warranted by the use of opaque envelopes.

Prior to SD (baseline), at 3, 6 and 12 months after SD the following parameters were assessed using a manual periodontal probe (PCPUNC 15, Hu Friedy, Chicago, IL, USA): FMPS [66], bleeding on probing (BOP) [68], probing pocket depth (PPD) and clinical attachment level (CAL). Additionally, furcation involvement (FI) was also measured using a manual furcation probe (Nabers probe -PQ2N, Hu Friedy, Chicago, IL, USA). Sessions including oral hygiene instruction and supragingival cleaning were performed until patients reached a FMPS ≤ 25%. All clinical recordings and oral hygiene instructions were performed by one single examiner (RJ) blinded to treatment allocation. Subsequently, SRP was performed within 2 consecutive days by one experienced periodontist (RC) also blinded to group allocation. All sites with PD≥ 4mm were scaled and root-planed to the bottom of the pocket with machine driven scalers (Kavo Sonicflex Scaler, Kavo Dental GmbH, Biberbach, Germany) and Gracey curets (Hu Friedy, Chicago, IL, USA). Treated pockets were then thoroughly rinsed with 0.12% chlorhexidine digluconate solution. For 14 days, subjects rinsed with a 0.2% chlorhexidine digluconate solution (Corsodyl^®^, GlaxoSmithKline, Brentford, London, UK, twice daily for 2 minutes) and brushed their teeth with 0.2% chlorhexidine digluconate tooth paste (Elugel^®^, Pierre Fabre, Paris, France). At the end of the SD sessions, patients were allocated by another clinician (RT) to one of the three treatment groups, received their medications and intake instructions (for detailed description about the bottle pills see [11]). Two weeks following SD, patients were asked about compliance with study medication and possible adverse effects after pills intake. Subsequently, patients were recalled at 3, 6 and 12 months when the following parameters were assessed: FMPS, BOP, PD, CAL, FI. At these appointments, only supragingival calculus was removed leaving any residual periodontal pockets with PD = 4 mm and BOP or PD≥5 mm without re-instrumentation.

Five patients, each with minimum 10 teeth with at least one site with PD ≥ 6 mm per tooth, were used to calibrate the examiner. Patients were examined twice on 2 appointments, 48 hours apart. Calibration was accepted when these measurements were similar to the millimetre at >90% (mean intra-examiner reliability: PD: 0.87, CAL: 0.76, Cohen`s Kappa Analyses). The examiner was blinded to the treatment allocation.

### Statistical analyses

All data were introduced into a database (LI) and double-checked for errors by comparison with the original patients’ charts (RT, RC). Statistical analyses were performed by an experienced professional statistician (CH) using the statistical software programs (SPSS statistics 21, IBM, Armonk, NY, USA and R Core Team, 2016. R: A language and environment for statistical computing. R Foundation for Statistical Computing, Vienna, Austria).

The statistical unit was the patient; the primary outcome variable was the reduction (Δ) in the number of sites per patient with PD ≥ 6 mm calculated between baseline and 12 months. Secondary variables were mean changes in FMPS, BOP, PD, AL, number of sites with PD ≥ 6 mm, with PD ≥ 5 mm and total number of sites with PD = 4 positive on probing and sites with PD≥5mm at 12 months, as well as the number of subjects reaching a low risk for disease progression (≤4 sites with PD≥5mm) [69]. Statistically significant were considered *p-*values < 0.05.

All sites with PD≥ 4 mm at baseline were considered for statistical analyses. Intra-group clinical comparisons between the follow-ups were analysed by means of Paired t-Test and Wilcoxon Signed Ranks Test. Comparisons between the groups at the various timepoints were performed adjusting for baseline values and smoking using ANCOVA and Bonferroni adjustments.

The residual number of sites with PD≥5 mm was analysed and according to it patients were distributed to low (≤4 sites), moderate (5–8 sites) and high (≥9 sites) risk for disease progression [69]. Group comparisons were performed using Fischer`s Exact Test.

By means of a Poisson regression analysis, the relationship between the number of sites with PD ≥ 6 mm at 12 months and the variables treatment (antibiotics for 3 or 7 days), smoking, gender, BOP, FMPS, at baseline and at 12 months, baseline mean PD, baseline mean AL, disease severity with ≥10 sites with PD ≥ 6 mm at baseline was studied.

## Results

### Subjects

In total, 102 subjects (mean age 43.37 ± 9.85, 65 female, 35 smokers) fulfilling the inclusion criteria (34 per treatment group) were enrolled in this study and were treated and followed-up according to the protocol between January 2012 and June 2015. Considering the dropouts during the three follow-ups, 27 patients were excluded from the final *per protocol* (PP) analyses. Reasons for dropout were: non-compliance with the appointments (n = 16), antibiotic intake for other medical reasons (n = 9), moved to another town (n = 2). Eventually, 75 subjects [mean age 42.59 ± 9.68 years, 45 female (60%), 22 smokers (29.33%)] were included in the final analyses (Fig 1). Patient distribution resulted in no statistically significant differences between the groups regarding gender, smoking status and baseline clinical parameters (Tables 1 and 2). Patient compliance with the pills intake was assessed at two weeks after SD and outlined excellent compliance with only some minor time-interval differences [11]. Adverse events occurring during or after the medication period were registered in all treatment groups: 1–2 patients in each treatment group had headaches, musculoscheletal pain, taste disorders and vertigo; 1 patient in placebo- and in group B complained about gastrointestinal disorders, while I patient in placebo and 1 in group C had fever. Only 1 patient in group B had respiratory disorders and I patient in the placebo group complained about shivering. None of the patients had signs of allergy nor candida [11].

**Table 1:** Baseline demographic characteristics (patients included in the PP analysis, PD and CAL values for all sites).

Variables	Group A	Group B	Group C
	N = 26	N = 24	N = 25
**Female gender (n/%)**	16/61.5	15/62.5	14/56
**Age (years)**	41.84±10.04	42.37±9.87	43.56±9.46
**Smoker (%)**	8	8	6
**BOP(%)**	62.89±27.80	58.89±35.94	71.05±24.39
**FMPS (%)**			
**Recruition timepoint**	54.29±20.93	52.53±22.46	47.46±23.35
**Baseline after OH instructions**	20.37±6.43	17.44±7.21	19.03±5.58
**Mean PD (mm)** ^**♯**^	4.54±0.78	4.58±0.72	4.61±0.61
**Mean CAL (mm)** ^**♯**^	5.17±1.26	5.13±1.29	5.13±1.12
**No. (%) PD≤4mm**	41.88±17.31 (52.65±13.66)	47.71±18.82 (51.38±12.97)	47.16±18.87 (45.98±14.26)
**No. (%) PD 4-6mm**	41.88±17.31 (52.65±13.66)	47.71±18.82 (51.38±12.97)	47.16±18.87 (45.98±14.26)
**No. (%) PD≥7mm**	12.81±12.91 (15.33±12.67)	12.96±8.93 (14.87±9.16)	19.20±12.84 (17.75±9.75)

PD = pocket depth, CAL = clinical attachment level, BOP = bleeding on probing; FMPS = full-mouth plaque score after O’Leary [66]

^♯^total mean values calculated for all sites, including values <4mm at baseline

**Table 2:** Mean values and group comparisons (ANOVA adjusted for smoking and ANCOVA adjusted for baseline values and smoking for 12 month variables), and their changes (Δ) between baseline and 12 months (mean±SD) (PP analyses, intragroup comparisons between baseline and 12 m by means of Paired t-Test and Wilcoxon Signed Ranks Test).

Variables	Group A (SRP+Placebo)N = 26	Group B (SRP+AB 3d) N = 24	Group C (SRP+AB 7d) N = 25	Group comparisons			Smoker—Non-smoker *P value*
				*p* value groups A-B	*p* value groups A-C	*p* value groups B-C	
**Δ No. PD≥6 mm**							
**baseline- 12m**	20.31±15.81 ^hs^	27.71±15.00 ^hs^	31.40±17.67 ^hs^	0.313	0.061	0.100	0.226
**PD (mm)**							
Baseline	5.43±0.61	5.43±0.51	5.75±0.59	1.000	0.180	0.196	0.197
12 m	3.31±0.42^hs^	2.91±0.46 ^hs^	3.00±0.56 ^hs^	0.011^s^	0.047^s^	1.000	0.034^s^
**Δ baseline- 12m**	2.04±0.71	2.55±0.65	2.67±0.80	0.057	0.012^s^	0.843	0.016^s^
**CAL (mm)**							
Baseline	5.91±1.26	5.82±1.23	6.10±1.25	1.000	1.000	1.000	0.640
12 m	4.67±1.18 ^s^	4.26±1.23 ^hs^	4.29±1.17 ^hs^	0.317	0.451	0.959	0.004^s^
**Δ baseline- 12m**	1.20±0.73	1.59±0.65	1.71±0.54	0.093	0.017^s^	0.789	0.009^s^
**BoP (%)**							
Baseline	62.83±27.80	58.89±35.94	71.05±24.39	1.000	0.981	0.473	0.929
12 m	12.91±10.01 ^hs^	10.08±7.13 ^hs^	10.95±9.16 ^hs^	0.769	1.000	1.000	0.480
**Δ baseline-** **12m**	51.92±25.51	48.81±36.24	60.10±28.23	1.000	1.000	0.585	0.852
**FMPS (%)**							
Baseline	20.37±6.43	17.44±7.21	19.03±5.58	0.352	1.000	1.000	0.209
12 m	20.74±12.93	33.49±24.21^s^	30.01±19.87^s^	0.032	0.256	1.000	0.097
**Δ baseline- 12m**	-0.94±12.23	-16.08±22.92	-10.98±19.99	0.020	0.227	0.914	0.120
**PD 4–6 mm (mm)**							
Baseline	4.88±0.22	4.89±0.22	4.96±0.22	1.000	0.611	0.846	0.880
12 m	3.06±0.36	2.74±0.39	2.77±0.48	0.016^s^	0.047^s^	1.000	0.007^s^
**Δ baseline- 12m**	1.82±0.39	2.15±0.46	2.19±0.50	0.027^s^	0.018^s^	1.000	0.018^s^
**CAL of PD 4–6 mm (mm)**							
Baseline	5.49±1.13	5.32±1.17	5.40±1.06	1.000	1.000	1.000	0.217
12 m	4.41±1.06	4.03±1.23	3.95±1.15	0.282	0.031^s^	1.000	0.013 ^s^
**Δ baseline- 12m**	1.07±0.59	1.29±0.52	1.46±0.44	0.382	0.039^s^	1.000	0.027^s^
**PD >7 mm (mm)**							
Baseline	7.60±0.54	7.61±0.67	7.82±0.88	1.000	0.826	0.910	0.779
12m	4.44±1.06	3.60±0.93	3.70±0.96	0.009^s^	0.043^s^	1.000	0.011^s^
**Δ baseline- 12m**	3.15±1.26	4.01±0.96	4.12±1.49	0.036^s^	0.016^s^	1.000	0.193
**CAL of PD >7 mm (mm)**							
Baseline	7.70±0.92	7.96±1.54	7.79±1.41	1.000	1.000	1.000	0.110
12 m	5.62±1.29	5.34±1.65	5.26±1.46	0.110	0.405	1.000	0.037^s^
**Δ baseline- 12m**	1.98±1.21	2.67±1.12	2.42±0.79	0.065	0.424	1.000	0.101
**No. PD≥6mm**							
Baseline	25.50±17.36	29.37±14.65	35.92±17.58	1.000^s^	0.098	0.592	0.408
12 m	5.19±3.71 ^hs^	1.66±2.26 ^hs^	4.52±5.40 ^hs^	0.003^s^	0.917	0.068	0.093
**No. PD≥5mm**							
Baseline	41.73±19.91	46.21±18.65	53.24±20.65	1.000	0.129	0.665	0.989
12 m	10.50±6.85	4.71±5.40	8.44±8.92	0.004^s^	0.453	0.202	0.003^s^
**Δ baseline- 12m**	31.23±17.48	41.50±18.67	44.80±21.37	0.180	0.050	1.000	0.269
**No. PD = 4 mmBOP+ and PD≥5mm**							
Baseline	54.69±22.84	60.66±22.345	66.36±23.24	1.000	0.227	1.000	0.965
12 m	12.34±8.15	6.41±6.25	10.97±8.94	0.012^s^	1.000	0.153	0.002^s^
**Δ baseline- 12m**	42.34±20.85 ^hs^	54.25±21.68 ^hs^	55.44±23.97 ^hs^	0.174	0.137	1.000	0.229

PD = pocket depth, CAL = clinical attachment level, BOP = bleeding on probing; FMPS = full-mouth plaque score after O’Leary [66], m: months, base: baseline, PP = per protocol analyses

^s^ statistically significant *p<0*.*05* values

^hs^ statistically highly significant *p<0*.*0001*.

### Clinical findings

Clinical findings at 3 and 6 months after non-surgical periodontal treatment were reported in a previous publication [11]. At 6 months, 91 of the 102 included subjects completed the evaluation and statistically significant clinical improvements (p<0.05) compared to baseline were observed in all treatment groups for all evaluated clinical parameters. These statistically significant improvements were maintained up to one year in all three groups, even if fewer subjects (75 out of 102 subjects) completed the study (p<0.05, paired t-Test and Wilcoxon Signed Ranks Test). At baseline, no statistically significant differences between the treatment groups were registered for any of the investigated clinical parameters (Tables 1 and 2, p>0.05). BOP was statistically significantly reduced in all treatment groups compared to baseline. However, no statistically significant differences between the groups were seen at baseline and at 12 months. In all three groups, FMPS shoved a statistically insignificant increase at 12 months compared to baseline, without any inter-group differences (Table 2).

At 12 months, both antibiotic groups resulted in statistically significant improvements compared to the placebo group regarding the total mean PD as well as the mean PD and PD reductions (Δ base-12m) for sites with PD 4-6mm and deep sites with PD≥7mm (Table 2). Total mean PD reductions (Δ base-12m) were significantly higher only in group C compared to group A. CAL gain (Δ base-12m) at 12 months was significantly higher in the 7-day AB group compared to placebo for all sites with PD≥4mm as well as for sites with PD 4-6mm. However, for sites with PD≥7mm no statistically significant differences were observed between the three groups. Furthermore, at 12 months, the 3-day AB group showed significantly less sites with PD ≥6mm, with PD≥5mm and with PD = 4mm and BOP+ compared to the control group (Table 2). Nonetheless, the reduction in the number of sites with PD≥6 mm (the primary outcome variable), showed no statistical differences between the three patient groups. No statistically significant changes were seen for any of the evaluated clinical parameters between the two antibiotic groups (p>0.05).

At 12 months, smokers exhibited statistically significantly less improvements compared to non-smokers with respect to mean PD and significantly less PD reductions, mean CAL and mean CAL gain in all PD categories (p<0.05, Table 2). For sites with PD≥7mm, smokers had significantly higher mean PD and CAL at 12 months; however there were no statistically significant differences for PD reduction and CAL gain (p>0.05, Table 2). Moreover, smokers showed less sites with PD≥5mm and with PD≥4mm and BOP+ (p<0.05, Table 2).

Analysing the risk for disease progression [69], all patients in all treatment groups were at baseline at high risk (Table 3). At all follow-ups a decrease was noticed in all treatment groups, so that at 12 months the lowest percentage (20.8%) of patients being at high risk for disease progression was observed in the 3-day AB group, followed by the 7-day AB group with 40% and finally by the placebo group with 46.2%. Comparing the groups regarding the number of subjects that reached a low risk of disease progression presenting ≤4 sites with PD≥5mm, more patients were present in both antibiotic groups as compared to the placebo group (Table 3). However, in the PP-analyses only the 3-day AB group exhibited significantly more patients with low risk at 12 months compared to the placebo group with no significant differences between groups B and C (Table 4). The difference between groups A and C was only present in the ITT population (p = 0.049). A Bonferroni correction (3 tests comparing groups A-B, A-C, B-C) would lead to significant differences only between groups A and B (ITT and PP).

**Table 3:** Patients (Number and %) with low (≤4 number of residual sites with PD≥5mm ≤4), moderate (Number of residual sites with PD≥5mm: 5–8) and high (Number of residual sites with PD≥5mm ≥9) risk for disease progression [69].

Risk categories	Group A N = 26 (PP) N = 27 (ITT)	Group B N = 24 (PP) N = 26 (ITT)	Group C N = 25 (PP) N = 27 (ITT)	A–B Fisher-Test	A–C Fisher-Test	B–C Fisher-Test
**ITT (n = 80)**						
**Baseline**				-	-	-
**≤4 sites PD≥5mm (n/%)**	0	0	0			
**5–8 sites PD≥5mm (n/%)**	0	0	0			
**≥9 sites PD≥5mm (n/%)**	30/100	30/100	31/100			
**3 months**				0.001^s^	0.314	0.053
**≤4 sites PD≥5mm (n/%)**	2/7.1	15/50	6/21.4			
**5–8 sites PD≥5mm (n/%)**	6/21.4	6/20	6/21.4			
**≥9 sites PD≥5mm (n/%)**	20/71.4	9/30.0	16/57.1			
**6 months**				<0.001^s^	0.023^s^	0.036^s^
**≤4 sites PD≥5mm (n/%)**	2/6.7	18/66.7	11/35.5			
**5–8 sites PD≥5mm (n/%)**	8/26.7	1/3.7	6/19.4			
**≥9 sites PD≥5mm (n/%)**	20/66.7	8/29.6	14/45.2			
**12 months**				<0.001^s^	0.091	0.264
**≤4 sites PD≥5mm (n/%)**	6/21.4	18/72.0	13/48.1			
**5–8 sites PD≥5mm (n/%)**	8/32.1	2/8.0	4/14.8			
**≥9 sites PD≥5mm (n/%)**	13/46.4	5/20.0	10/37.0			
**PP (n = 75)**						
**Baseline**				-	-	-
**≤4 sites PD≥5mm (n/%)**	0	0	0			
**5–8 sites PD≥5mm (n/%)**	0	0	0			
**≥9 sites PD≥5mm (n/%)**	26/100	24/100	25/100			
**3 months**				0.005 ^s^	0.146	0.262
**≤4 sites PD≥5mm (n/%)**	2/7.7	11/45.8	6/24.0			
**5–8 sites PD≥5mm (n/%)**	4/15.4	4/16.7	6/24.0			
**≥9 sites PD≥5mm (n/%)**	20/76.9	9/37.5	13/52.0			
**6 months**				<0.001^s^	0.004^s^	0.280
**≤4 sites PD≥5mm (n/%)**	1/3.8	15/62.5	10/40.0			
**5–8 sites PD≥5mm (n/%)**	8/30.8	1/ 4.2	3/ 12.0			
**≥9 sites PD≥5mm (n/%)**	17/65.4	8/33.3	12/48.0			
**12 months**				0.001^s^	0.120	0.164
**≤4 sites PD≥5mm (n/%)**	5/19.2	17/70.8	11/44.0			
**5–8 sites PD≥5mm (n/%)**	9/34.6	2/8.3	4/16.0			
**≥9 sites PD≥5mm (n/%)**	12/46.2	5/20.8	10/40.0			

ITT: Intention to treat analyses, PP: per protocol analyses, PD: pocked depth, m: months

**Table 4:** Patients reaching a low risk of disease progression (≤4 sites with PD≥5 mm) at 12 months.

Timepoints	Group A ITT: n = 28 PP: n = 26	Group B ITT: n = 25 PP: n = 24	Group C ITT: n = 27 PP: n = 25	Groups A-B (Fisher-Test)	Groups A-C (Fisher Test)	Groups B-C (Fisher Test)
**ITT analysis (n = 80)**	6/21.4%	18/72.0%	13/48.1%	<0.001^s^	0.049^s^	0.097
**PP analysis (n = 75)**	5/19.2%	17/70.8%	11/44.0	<0.001^s^	0.070	0.080

ITT: Intention to treat analyses, PP: per protocol analyses, PD: pocked depth, m: months

At 12 months, the regression analysis showed a statistically significant positive correlation between the residual number of sites with PD≥6mm and the placebo group (group B had 0.28 and group C 0.70 times the expected number of residual sites with PD≥6mm at 12 months than group A), male gender and smokers. Further significant variables were percentage of BOP at baseline and at 12 months, baseline elevated level of BOP and initial mean CAL (Table 5).

**Table 5:** Poisson regression analyses for the number of residual sites with PD ≥ 6 mm at 12 months after non-surgical periodontal therapy as related to placebo group (group A).

Variables	Exponential Coefficient (e^B^)	95% CI	*p* value
Group B (ABAb for 3 d)	0.286	0.193–0.424	<0.001^s^
Group C (ABAb for 7 d)	0.7023	0.526–0.939	0.017^s^
Female gender	0.6690	0.537–0.887	0.004^s^
Smoker	1.364	1.057–1.761	0.017^s^
≥10 sites with PD≥6 mm at baseline	1.295	0.837–2.004	0.246
BOP baseline	0.995	0.990–0.999	0.044^s^
BOP at 12m	1.027	1.013–1.041	<0.001 ^s^
FMPS baseline	0.997	0.991–1.002	0.244
FMPS at 12m	1.000	0.989–1.010	0.933
GBI baseline	0.997	0.961–0.003	0.004
GBI at 12m	0.986	0.973–1.000	0.056
Mean PD baseline	0.890	0.664–1.192	0.433
Mean CAL baseline	1.248	1.077–1.447	0.003^s^

Ab = antibiotics, PD = pocket depth, CAL = clinical attachment level, BOP = bleeding on probing, GBI: gingival bleeding index [68]^s^ statistically significant *p< 0*.*05*. Reference categories are: Group A, Male, Non-Smoker

## Discussion

The present study evaluated the 12 months clinical outcomes following a 3 or 7 days systemic administration of AMX and MET adjunctive to SD as compared with non-surgical periodontal treatment alone in patients with severe ChP. A 3-day systemic administration of AMX and MET yielded comparable clinical outcomes as a 7-day AMX and MET regimen as an adjunct to SD.

Since several clinical trials have evaluated different administration periods for antibiotics adjunctive to non-surgical periodontal therapy, like of 7 [19, 22, 29, 70] to 14 days [17, 26, 27, 71] and no consensus regarding the optimal antibiotic regimen exists, the aim of the present study was to provide additional data for determining the optimal antibiotic dosage and duration adjunctive to the non-surgical periodontal treatment. Bearing in mind the fact that possible emergence of new resistant strains may occur during or after a single long-term infection associated with antibiotic therapy and considering the widespread use of antibiotics in our society, antimicrobial resistance became nowadays a global burden [49, 50]. As several areas in general medicine succeeded in restricting and optimizing as much as possible the antibiotic regimen by reducing the duration of antibiotic intake and increasing the dosage [55–57, 63, 72] it seems appropriate that similar measures should be taken in dentistry, restricting antibiotic treatment to patients with the most benefit from it and setting an optimal treatment regime (minimum bactericidal concentration and duration) [37]. Moreover, this should be one of the main aims in research since a significant increase in the proportion of resistant strains to the most common used antibiotics in dentistry, especially to AMX and MET which have been shown to be most effective in treating severe forms of periodontitis, had already been registered [73–79]. Considering these aspects and knowing that the dissemination of periodontal pathogens in other body parts may lead to serious life-threatening diseases (such as brain abscesses, lung infections, endocarditis, soft-tissue infections) [80], it seems of outmost importance to optimize the adjunctive use of antibiotics in the treatment of periodontal disease.

While several studies already exist in evaluating and comparing dosages of 250-500mg of AMX and MET adjunctive to non-surgical periodontal treatment and durations of 7–14 days [15, 17–19, 22, 26, 27, 29, 32, 71, 81, 82] and showing significant clinical and partly microbiological benefits in the antibiotic groups over the mechanical therapy alone, we found it relevant to evaluate the effectiveness of a short-treatment antibiotic course of 3 days [11]. However, this duration might be an issue concerning the development of antimicrobial resistance. Nonetheless, we decided to test this antibiotic duration based on the observations from the study of Feres et al. [53], where a transient increase of the bacterial resistance was shown to be lower in the 3 day as compared to the 7 day antibiotic regimen. Moreover, favourable results were obtained for short-treatment courses of acute streptococcal pharyngitis in children [83, 84], for acute urinary tract infections [85], intra-abdominal infections and infections in general surgery units [86, 87].

In the present study at 12 months, all treatment groups showed statistically significant reductions (Δ) in the number of sites with PD≥6 mm compared to baseline (p<0.05) (primary outcome variable, Table 2) [11]; no statistically significant differences could be detectable between the three treatment groups, even though the reductions were higher in the two antibiotic groups compared to the control group (Table 2). This may be explained by the fact that in the present study at 12 months, a higher attrition in the number of patients compared to the priory calculated study power was observed in all treatment groups (below 30 patients/group at 12 months) resulting in low power assessments for the primary outcome (post hoc analysis: for comparison group A-B 35%, for A-C 64%). Wide deviation ranges for the number of sites with PD≥6mm at baseline (14. 65–17.58 sites) may have also influenced the fact that no significant differences between the 3 groups were obtained for the their reductions (Δ). However, at 12 months, only the 3-day antibiotic group showed statistically significantly less number of residual deep sites (PD≥6mm), as compared to the placebo group (p = 0.003), even though the placebo group showed at baseline less deep sites (25.5±17.36) compared to the 2 antibiotic groups (3 days AB 29.37±14.65, 7 days AB 35.92±17.58, Table 2). Nonetheless, since the number of residual deep sites (PD≥6 mm) was a secondary outcome variable, these results have to be interpreted with caution. Similar results were obtained also for the residual number of sites with PD≥5mm or with PD≥4mm and BOP+, where only the 3-day AB group showed statistically significantly less sites compared to placebo. The fact that in the 7-day antibiotic group the residual number of such sites (with PD≥6mm, PD≥5mm, PD≥4mm and BOP+) was not statistically significantly lower compared to the placebo group might be related to the higher number of such sites at baseline in group C as compared to group A [for PD≥6mm: placebo 25.50 ± 17.36 (mean±SD), 7 day AB group 35.92±17.58(mean±SD)], even if no statistically significant differences were obtained between the three treatment groups at baseline (Table 2).

Our 12-month findings corroborate those obtained by Feres et al., the 3-day AB group in our study showing comparable residual number of sites with PD≥6mm (1.66±2.26) to the 14-day AMX and MET group of Feres et al. (1.2±2.2) [27]. Comparable results for deep sites were also registered by Harks et al., where patients receiving AMX and MET for 7 days showed at 27.5 months 0.9±1.6% of sites with PD≥7mm [81] and by Rooney et al. at 6 months (the AMX+MET group had 1.3±2.6% of sites with PD≥6mm) [16]. Furthermore, looking at the number of sites with PD≥5mm our data are similar to those obtained by Feres et al. at 12 months (Feres et al. AMX+MET group: 4.7±6.0, our study 3-day AB group: 4.71±5.4 sites) and Silva et al. obtained at 3 months (Silva et al. AMX+MET for 14 days 5.3±4.4) [26, 27]. However, Cionca et al. showed at 6 months that the AMX+MET group had a residual number of sites with PD>5mm and BOP+ of 0.4±0.8 [19] much lower than the number of such sites in both AB groups from our study (group B: 6.41±6.25, group C: 10.97±8.94). As reported previously, baseline periodontal conditions of the present study differ from those of Cionca et al. who included patients with moderate to severe ChP with at least four teeth with PD > 4mm and CAL ≥ 2 mm as opposed to generalised severe forms of ChP included in the present study [11, 19]. This is reflected in the baseline number of sites with PD ≥ 5 mm, which was at least double as high in every treatment group of the present study compared to the corresponding values in the study by Cionca et al. [19] as well as by the fact that all patients in the present study had at baseline over 9 sites with PD≥5mm (Table 3).

All three treatment groups obtained at 12 months statistically significant PD reductions and CAL gain as compared to baseline. Nonetheless, the mean PD values at 12 months were significantly lower in both AB groups as compared to placebo (p<0.05, Table 2). Mean PD reductions and CAL gain were however statistically significantly higher only in the 7-day AB group as compared to placebo. This might be due to the fact that this group presented at baseline, even if statistically insignificant, deeper sites as compared to the other two groups (group A and B) as seen from the baseline mean values for every PD category (total mean PD, PD 4-6mm, PD>7mm, Table 2). Furthermore, the higher number of deep sites (PD≥7mm) at baseline in the 7-day antibiotic group, even if statistically insignificant when compared to the other two groups, might have influenced the outcomes for PD reductions and CAL gain, considering that deeper sites might benefit more from an AB regimen compared to more shallow ones [15, 19, 26, 27, 32]. However, PD reductions for sites with PD between 4-6mm and for sites with PD≥7mm were statistically significantly higher in both AB groups as compared to the placebo group, with no statistically significant differences when comparing the 3- and the 7-day AB groups (Table 2). Comparable results in terms of mean PD, were obtained by Feres et al. at 12 months (placebo: 3.05±0.59mm, AMX+MET for 14d: 2.54±0.40mm), by Cionca et al. at 6 months (placebo: 3.1±0.3mm, AMX+MET for 7 days 3.0±0.2mm) [19, 27]. Harks et al. obtained at 27.5 months in both treatment groups slightly lower mean PD values as opposed to the ones in the present study (Placebo: 2.6±0.7mm, AMX+MET for 7 days: 2.3±0.5mm) [81], these differences being possibly explained by the baseline mean PD values, which were about 2mm lower than those in our study (Table 2) and by the fact that they reported results of a twice longer follow-up (27.5 months) compared to our study (12 months). Furthermore, mean PD reductions obtained in our study in all treatment groups were similar to those obtained in the SRP and AB groups of Goodson et al. at 24 months (SRP alone: 1.81±0.23mm, SRP +AMX+MET for 14 days: 2.36±0.2 mm) [71]. Moreover, CAL gain in the AB group of Goodson et al. (1.53±0.16 mm) was also comparable to that obtained in our study (3-day AB group: 1.59±0.65, 7-day AB group: 1.71±0.54 mm). Slightly less CAL gain was observed by Cionca et al., Feres et al. and Harks et al. [19, 27, 81]. These differences might be attributed to the approximately 2 mm higher baseline CAL values in the patients included in the present study as compared to those in the aforementioned studies.

Studies determining the clinical efficacy of a treatment use diverse criteria for defining success [88] such as changes of PD and CAL, number and proportions of moderate to deep sites [15, 16, 19, 27, 81]. Since ChP is an inflammatory disease initiated by bacterial infection, the most important part of its therapy is the treatment of infection and elimination of inflammation to arrest disease progression and improve tooth prognosis. The use of full-mouth PD and CAL as outcome variables in several studies [15, 17, 26, 27, 32] evaluating the effectiveness of antibiotic treatment in non-surgical periodontal therapy may not be representative enough for the effectiveness of an antibiotic treatment, since shallow sites have a lesser adjunctive benefit from antibiotics as opposed to deep sites [15, 19, 26, 27, 32], and may not emphasize the treatment effect for deep sites which have been shown to be at high risk for disease progression [64, 69, 89]. In this sense, mean PD and CAL values as well as their reductions/gain may not be the most appropriate parameters for assessing the elimination of inflammation and effectiveness of a treatment.

Since disease progression is more likely to occur in deep sites, it seems relevant to evaluate the residual number of such sites or changing the patient attribution from a severe to low risk of disease progression. Considering this, we analysed both the residual number of deep sites (PD≥6 mm) and patient attribution to risk for disease progression [69]. Irrespective of the group allocation, all patients presented at baseline high risk for disease progression. After treatment, the percentage of these patients decreased continuously in all groups up to 12 months. A closer analysis of the risk distribution among groups revealed that almost half of the patients in the placebo group were still at high risk for progression, while the 3-day AB group showed the lowest number of patients with high risk. Finally, 70% of the patients in the 3-day AB group reached a clinical endpoint of low disease progression (<4 sites with PD≥5 mm), followed by the 7 days AB group with over 40% and by the placebo group with only 20%, which was statistically significantly lower only compared to the 3-day AB group (PP analyses). Furthermore, similar percentages for groups A and C were obtained for the number of patients that maintained a high risk for disease progression (≥9 sites with PD≥5 mm) and more sites PD≥5mm at 12 months. The fact that group C didn`t obtain in the PP-analyses significantly less patients at low risk compared to group A may be related to the higher number of sites with PD≥5mm and sites with PD≥6mm at baseline, even if this difference didn`t reach statistical significance. These values are comparable to those obtained in studies investigating this parameter: Feres et al. at 12 months (Placebo: 22.5%, AMX+MET: 66.7%), Harks et al. at 27.5 months (Placebo 36.5%, AMX+MET: 63.1%) [27, 81].

In the present study, smokers exhibited at 12 months statistically significantly lower clinical improvements as compared to non-smokers, and this was observed for most of the evaluated parameters (Table 2). This is in line with data from other studies evaluating non-surgical periodontal treatment that showed significantly lower clinical improvements in smokers [15, 90–92]. Moreover, this is outlined in the results of the Poisson regression analyses (Table 4), where smoking together with antibiotics (both 3- and 7-day antibiotic regimen), female gender, BOP at baseline and 12 months, initial gingival marginal bleeding (GBI) and initial CAL statistically influenced the residual number of sites with PD≥6mm at 12 months. This is in agreement with other studies, where antibiotic treatment was found to be the only variable that significantly influenced the clinical outcome [19, 27].

The trend for better clinical outcomes after 7 days of AMX and MET observed at 6 months [11] was maintained at 12 months. As mentioned earlier and in our previous publication, we sized the study with 30 subjects per treatment group in order to detect a difference of 5 sites with PD≥6 mm [19, 27, 64] for a power of 90% (significance level 0.05). Since the present study was designed as a superiority trial of the two antibiotic protocols over the mechanical debridement alone, it cannot be ruled out that the direct comparison between the 3- and the 7-day protocol might be underpowered and therefore, this comparison should be interpreted with caution. Thus, based on the present results, no conclusions regarding the direct comparison between the 3- and the 7-day antibiotic protocol can be drawn. Obviously, in order to adequately address this important issue, future prospective, randomized, controlled clinical studies with an appropriate design and including sufficient number of patients need to be conducted.

A limitation of the present study represents the high attrition rate below 30 (according to the initial study power calculation) recommending a careful interpretation of the present results. The high dropout rate in all 3 groups was due to the facts that several patients either omitted their follow-up appointments, have taken antibiotics in the follow-up period for other medical reasons, or were further surgically treated (i.e. dental implants) by their referring dentists. The finding that at 12 months, the study has failed to reveal statistically significant differences for the main outcome variable between any of the treatment groups, may, on one hand, be related to the high attrition rate leading to a low study power at 12 months. However, on the other hand, wide ranges for the standard deviation of the number of deep sites (PD≥6mm) at baseline may have also influenced the results. Nonetheless, it has to be pointed out that the majority of the investigated variables revealed statistically significantly better clinical improvements (Table 2.) in both antibiotic groups compared to the placebo group, but these findings need to be carefully interpreted being secondary outcomes.

In conclusion, the present study has shown that: a) in patients with severe chronic periodontitis non-surgical periodontal therapy in conjunction with either three or seven days systemic administration of amoxicillin (AMX) and metronidazole (MET) yields statistically significantly greater clinical improvements compared to non-surgical therapy alone.

## Supporting information

S1 Table ITT analyses: Mean values and group comparisons (ANCOVA adjusted for baseline values and smoking), and their changes (Δ) between baseline and 12 months (mean±SD). ITT = intention to treat, PD = pocket depth, CAL = clinical attachment level, BOP = bleeding on probing; FMPS = full-mouth plaque score after O’Leary [66], m: months, base: baseline. ^s^ statistically significant *p* values.
(DOCX)

S2 Table Raw data. (XLSX)

S1 Text Study protocol in English. (PDF)

S2 Text Study protocol in Romanian. (PDF)

## References

[1] Socransky SS, Haffajee AD. The bacterial etiology of destructive periodontal disease: current concepts. J Periodontol. 1992;63:322–31. doi: 10.1902/jop.1992.63.4s.322 29539682 10.1902/jop.1992.63.4s.322

[2] Mombelli A, Gmur R, Gobbi C, Lang NP. Actinobacillus actinomycetemcomitans in adult periodontitis. I. Topographic distribution before and after treatment. Journal of periodontology. 1994;65:820–6. doi: 10.1902/jop.1994.65.9.820 7990017 10.1902/jop.1994.65.9.820

[3] Mombelli A, Schmid B, Rutar A, Lang NP. Persistence patterns of Porphyromonas gingivalis, Prevotella intermedia/nigrescens, and Actinobacillus actinomyetemcomitans after mechanical therapy of periodontal disease. J Periodontol. 2000;71:14–21. doi: 10.1902/jop.2000.71.1.14 10695934 10.1902/jop.2000.71.1.14

[4] Renvert S, Wikstrom M, Dahlen G, Slots J, Egelberg J. Effect of root debridement on the elimination of Actinobacillus actinomycetemcomitans and Bacteroides gingivalis from periodontal pockets. Journal of clinical periodontology. 1990;17:345–50. 2204636 10.1111/j.1600-051x.1990.tb00029.x

[5] Dahlen G, Wikstrom M, Renvert S. Treatment of periodontal disease based on microbiological diagnosis. A 5-year follow-up on individual patterns. Journal of periodontology. 1996;67:879–87. doi: 10.1902/jop.1996.67.9.879 8884645 10.1902/jop.1996.67.9.879

[6] Grossi SG, Zambon JJ, Ho AW, Koch G, Dunford RG, Machtei EE, et al Assessment of risk for periodontal disease. I. Risk indicators for attachment loss. Journal of periodontology. 1994;65:260–7. doi: 10.1902/jop.1994.65.3.260 8164120 10.1902/jop.1994.65.3.260

[7] Haffajee AD, Socransky SS. Microbial etiological agents of destructive periodontal diseases. Periodontology 2000. 1994;5:78–111. 9673164 10.1111/j.1600-0757.1994.tb00020.x

[8] Carvalho LH, D'Avila GB, Leao A, Goncalves C, Haffajee AD, Socransky SS, et al Scaling and root planing, systemic metronidazole and professional plaque removal in the treatment of chronic periodontitis in a Brazilian population II—microbiological results. Journal of clinical periodontology. 2005;32:406–11. doi: 10.1111/j.1600-051X.2005.00720.x 15811059 10.1111/j.1600-051X.2005.00720.x

[9] Cugini MA, Haffajee AD, Smith C, Kent RL Jr., Socransky SS. The effect of scaling and root planing on the clinical and microbiological parameters of periodontal diseases: 12-month results. Journal of clinical periodontology. 2000;27:30–6. 10674959 10.1034/j.1600-051x.2000.027001030.x

[10] Keestra JA, Grosjean I, Coucke W, Quirynen M, Teughels W. Non-surgical periodontal therapy with systemic antibiotics in patients with untreated chronic periodontitis: a systematic review and meta-analysis. J Periodontal Res. 2015;50:294–314. doi: 10.1111/jre.12221 25142259 10.1111/jre.12221

[11] Cosgarea R, Juncar R, Heumann C, Tristiu R, Lascu L, Arweiler N, et al Non-surgical periodontal treatment in conjunction with 3 or 7 days systemic administration of amoxicillin and metronidazole in severe chronic periodontitis patients. A placebo-controlled randomized clinical study. J Clin Periodontol. 2016;43:767–77. doi: 10.1111/jcpe.12559 27027501 10.1111/jcpe.12559

[12] Haffajee AD, Socransky SS, Gunsolley JC. Systemic anti-infective periodontal therapy. A systematic review. Ann Periodontol. 2003;8:115–81. doi: 10.1902/annals.2003.8.1.115 14971252 10.1902/annals.2003.8.1.115

[13] Herrera D, Sanz M, Jepsen S, Needleman I, Roldan S. A systematic review on the effect of systemic antimicrobials as an adjunct to scaling and root planing in periodontitis patients. J Clin Periodontol. 2002;29 Suppl 3:136–59; discussion 60–2. 12787214 10.1034/j.1600-051x.29.s3.8.x

[14] van Winkelhoff AJ, Abbas F, Pavicic MJ, de Graaff J. Chronic conjunctivitis caused by oral anaerobes and effectively treated with systemic metronidazole plus amoxicillin. Journal of clinical microbiology. 1991;29:723–5. 1890173 10.1128/jcm.29.4.723-725.1991 PMC269860

[15] Winkel EG, Van Winkelhoff AJ, Timmerman MF, Van der Velden U, Van der Weijden GA. Amoxicillin plus metronidazole in the treatment of adult periodontitis patients. A double-blind placebo-controlled study. J Clin Periodontol. 2001;28:296–305. 11314884 10.1034/j.1600-051x.2001.028004296.x

[16] Rooney J, Wade WG, Sprague SV, Newcombe RG, Addy M. Adjunctive effects to non-surgical periodontal therapy of systemic metronidazole and amoxycillin alone and combined. A placebo controlled study. J Clin Periodontol. 2002;29:342–50. 11966932 10.1034/j.1600-051x.2002.290410.x

[17] Matarazzo F, Figueiredo LC, Cruz SE, Faveri M, Feres M. Clinical and microbiological benefits of systemic metronidazole and amoxicillin in the treatment of smokers with chronic periodontitis: a randomized placebo-controlled study. J Clin Periodontol. 2008;35:885–96. doi: 10.1111/j.1600-051X.2008.01304.x 18727657 10.1111/j.1600-051X.2008.01304.x

[18] Guerrero A, Griffiths GS, Nibali L, Suvan J, Moles DR, Laurell L, et al Adjunctive benefits of systemic amoxicillin and metronidazole in non-surgical treatment of generalized aggressive periodontitis: a randomized placebo-controlled clinical trial. J Clin Periodontol. 2005;32:1096–107. doi: 10.1111/j.1600-051X.2005.00814.x 16174275 10.1111/j.1600-051X.2005.00814.x

[19] Cionca N, Giannopoulou C, Ugolotti G, Mombelli A. Amoxicillin and metronidazole as an adjunct to full-mouth scaling and root planing of chronic periodontitis. J Periodontol. 2009;80:364–71. doi: 10.1902/jop.2009.080540 19254119 10.1902/jop.2009.080540

[20] Cionca N, Giannopoulou C, Ugolotti G, Mombelli A. Microbiologic testing and outcomes of full-mouth scaling and root planing with or without amoxicillin/metronidazole in chronic periodontitis. Journal of periodontology. 2010;81:15–23. doi: 10.1902/jop.2009.090390 20059413 10.1902/jop.2009.090390

[21] Ribeiro Edel P, Bittencourt S, Zanin IC, Bovi Ambrosano GM, Sallum EA, Nociti FH, et al Full-mouth ultrasonic debridement associated with amoxicillin and metronidazole in the treatment of severe chronic periodontitis. Journal of periodontology. 2009;80:1254–64. doi: 10.1902/jop.2009.080403 19656025 10.1902/jop.2009.080403

[22] Griffiths GS, Ayob R, Guerrero A, Nibali L, Suvan J, Moles DR, et al Amoxicillin and metronidazole as an adjunctive treatment in generalized aggressive periodontitis at initial therapy or re-treatment: a randomized controlled clinical trial. J Clin Periodontol. 2011;38:43–9. doi: 10.1111/j.1600-051X.2010.01632.x 21062335 10.1111/j.1600-051X.2010.01632.x

[23] Heller D, Varela VM, Silva-Senem MX, Torres MC, Feres-Filho EJ, Colombo AP. Impact of systemic antimicrobials combined with anti-infective mechanical debridement on the microbiota of generalized aggressive periodontitis: a 6-month RCT. Journal of clinical periodontology. 2011;38:355–64. doi: 10.1111/j.1600-051X.2011.01707.x 21303403 10.1111/j.1600-051X.2011.01707.x

[24] Mestnik MJ, Feres M, Figueiredo LC, Soares G, Teles RP, Fermiano D, et al The effects of adjunctive metronidazole plus amoxicillin in the treatment of generalized aggressive periodontitis: a 1-year double-blinded, placebo-controlled, randomized clinical trial. Journal of clinical periodontology. 2012;39:955–61. doi: 10.1111/j.1600-051X.2012.01932.x 22882646 10.1111/j.1600-051X.2012.01932.x

[25] Mestnik MJ, Feres M, Figueiredo LC, Duarte PM, Lira EA, Faveri M. Short-term benefits of the adjunctive use of metronidazole plus amoxicillin in the microbial profile and in the clinical parameters of subjects with generalized aggressive periodontitis. Journal of clinical periodontology. 2010;37:353–65. doi: 10.1111/j.1600-051X.2010.01538.x 20447259 10.1111/j.1600-051X.2010.01538.x

[26] Silva MP, Feres M, Sirotto TA, Soares GM, Mendes JA, Faveri M, et al Clinical and microbiological benefits of metronidazole alone or with amoxicillin as adjuncts in the treatment of chronic periodontitis: a randomized placebo-controlled clinical trial. J Clin Periodontol. 2011;38:828–37. doi: 10.1111/j.1600-051X.2011.01763.x 21762197 10.1111/j.1600-051X.2011.01763.x

[27] Feres M, Soares GM, Mendes JA, Silva MP, Faveri M, Teles R, et al Metronidazole alone or with amoxicillin as adjuncts to non-surgical treatment of chronic periodontitis: a 1-year double-blinded, placebo-controlled, randomized clinical trial. J Clin Periodontol. 2012;39:1149–58. doi: 10.1111/jcpe.12004 23016867 10.1111/jcpe.12004

[28] Varela VM, Heller D, Silva-Senem MX, Torres MC, Colombo AP, Feres-Filho EJ. Systemic antimicrobials adjunctive to a repeated mechanical and antiseptic therapy for aggressive periodontitis: a 6-month randomized controlled trial. Journal of periodontology. 2011;82:1121–30. doi: 10.1902/jop.2011.100656 21235333 10.1902/jop.2011.100656

[29] Yek EC, Cintan S, Topcuoglu N, Kulekci G, Issever H, Kantarci A. Efficacy of amoxicillin and metronidazole combination for the management of generalized aggressive periodontitis. J Periodontol. 2010;81:964–74. doi: 10.1902/jop.2010.090522 20214441 10.1902/jop.2010.090522

[30] Goutoudi P, Diza E, Arvanitidou M. Effect of periodontal therapy on crevicular fluid interleukin-1beta and interleukin-10 levels in chronic periodontitis. Journal of dentistry. 2004;32:511–20. doi: 10.1016/j.jdent.2004.04.003 15304296 10.1016/j.jdent.2004.04.003

[31] de Lima Oliveira AP, de Faveri M, Gursky LC, Mestnik MJ, Feres M, Haffajee AD, et al Effects of periodontal therapy on GCF cytokines in generalized aggressive periodontitis subjects. Journal of clinical periodontology. 2012;39:295–302. doi: 10.1111/j.1600-051X.2011.01817.x 22126282 10.1111/j.1600-051X.2011.01817.x PMC3373017

[32] Ehmke B, Moter A, Beikler T, Milian E, Flemmig TF. Adjunctive antimicrobial therapy of periodontitis: long-term effects on disease progression and oral colonization. J Periodontol. 2005;76:749–59. doi: 10.1902/jop.2005.76.5.749 15898936 10.1902/jop.2005.76.5.749

[33] Flemmig TF, Milian E, Karch H, Klaiber B. Differential clinical treatment outcome after systemic metronidazole and amoxicillin in patients harboring Actinobacillus actinomycetemcomitans and/or Porphyromonas gingivalis. Journal of clinical periodontology. 1998;25:380–7. 9650874 10.1111/j.1600-051x.1998.tb02459.x

[34] Rodrigues AS, Lourencao DS, Lima Neto LG, Pannuti CM, Hirata RD, Hirata MH, et al Clinical and microbiologic evaluation, by real-time polymerase chain reaction, of non-surgical treatment of aggressive periodontitis associated with amoxicillin and metronidazole. Journal of periodontology. 2012;83:744–52. doi: 10.1902/jop.2011.110333 22060046 10.1902/jop.2011.110333

[35] Shehab N, Patel PR, Srinivasan A, Budnitz DS. Emergency department visits for antibiotic-associated adverse events. Clinical infectious diseases: an official publication of the Infectious Diseases Society of America. 2008;47:735–43. 18694344 10.1086/591126

[36] Walker CB. Selected antimicrobial agents: mechanisms of action, side effects and drug interactions. Periodontology 2000. 1996;10:12–28. 9567935 10.1111/j.1600-0757.1996.tb00066.x

[37] Vogelman B, Craig WA. Kinetics of antimicrobial activity. J Pediatr. 1986;108:835–40. 3701535 10.1016/s0022-3476(86)80754-5

[38] Lopez NJ, Gamonal JA, Martinez B. Repeated metronidazole and amoxicillin treatment of periodontitis. A follow-up study. Journal of periodontology. 2000;71:79–89. doi: 10.1902/jop.2000.71.1.79 10695942 10.1902/jop.2000.71.1.79

[39] Loesche WJ, Grossman N, Giordano J. Metronidazole in periodontitis (IV). The effect of patient compliance on treatment parameters. Journal of clinical periodontology. 1993;20:96–104. 8436638 10.1111/j.1600-051x.1993.tb00336.x

[40] Nagy E, Urban E, Nord CE. Antimicrobial susceptibility of Bacteroides fragilis group isolates in Europe: 20 years of experience. Clinical microbiology and infection: the official publication of the European Society of Clinical Microbiology and Infectious Diseases. 2011;17:371–9. 10.1111/j.1469-0691.2010.03256.x 20456453

[41] Snydman DR, Jacobus NV, McDermott LA, Golan Y, Hecht DW, Goldstein EJ, et al Lessons learned from the anaerobe survey: historical perspective and review of the most recent data (2005–2007). Clinical infectious diseases: an official publication of the Infectious Diseases Society of America. 2010;50 Suppl 1:S26–33. 20067390 10.1086/647940

[42] Roberts SA, Shore KP, Paviour SD, Holland D, Morris AJ. Antimicrobial susceptibility of anaerobic bacteria in New Zealand: 1999–2003. The Journal of antimicrobial chemotherapy. 2006;57:992–8. doi: 10.1093/jac/dkl052 16507560 10.1093/jac/dkl052

[43] Karlowsky JA, Walkty AJ, Adam HJ, Baxter MR, Hoban DJ, Zhanel GG. Prevalence of antimicrobial resistance among clinical isolates of Bacteroides fragilis group in Canada in 2010–2011: CANWARD surveillance study. Antimicrobial agents and chemotherapy. 2012;56:1247–52. doi: 10.1128/AAC.05823-11 22203594 10.1128/AAC.05823-11 PMC3294939

[44] Lassmann B, Gustafson DR, Wood CM, Rosenblatt JE. Reemergence of anaerobic bacteremia. Clinical infectious diseases: an official publication of the Infectious Diseases Society of America. 2007;44:895–900. 17342637 10.1086/512197

[45] Fair RJ, Tor Y. Antibiotics and bacterial resistance in the 21st century. Perspect Medicin Chem. 2014;6:25–64. doi: 10.4137/PMC.S14459 25232278 10.4137/PMC.S14459 PMC4159373

[46] Prevention CfDCa. Antibiotic Resistance Threats in the United States, 2013. Available from: http://www.cdc.gov/drugresistance/threat-report-2013/.

[47] EuropeanMedicineAgency. The bacterial challange: time to react. A call to narrow the gap between multidrug-resistant bacteria in the EU and the development of new antibacterial agents: European Medicine Agency; 2013 Available from: http://ecdc.europa.eu/en/publications/Publications/0909_TER_The_Bacterial_Challenge_Time_to_React.pdf.

[48] Prevention. CfDCa. World Health Day: Media Fact Sheet. 2013. Available from: http://www.cdc.gov/media/release/2011/f0407_antimicrobialresistence.pdf.

[49] Lieberman TD, Michel JB, Aingaran M, Potter-Bynoe G, Roux D, Davis MR Jr., et al Parallel bacterial evolution within multiple patients identifies candidate pathogenicity genes. Nat Genet. 2011;43:1275–80. doi: 10.1038/ng.997 22081229 10.1038/ng.997 PMC3245322

[50] Musher DM, Dowell ME, Shortridge VD, Flamm RK, Jorgensen JH, Le Magueres P, et al Emergence of macrolide resistance during treatment of pneumococcal pneumonia. N Engl J Med. 2002;346:630–1. doi: 10.1056/NEJM200202213460820 11856810 10.1056/NEJM200202213460820

[51] Lipsitch M, Levin BR. The population dynamics of antimicrobial chemotherapy. Antimicrobial agents and chemotherapy. 1997;41:363–73. 9021193 10.1128/aac.41.2.363 PMC163715

[52] Barbosa TM, Levy SB. The impact of antibiotic use on resistance development and persistence. Drug resistance updates: reviews and commentaries in antimicrobial and anticancer chemotherapy. 2000;3:303–11. 11498398 10.1054/drup.2000.0167

[53] Feres M, Haffajee AD, Allard K, Som S, Goodson JM, Socransky SS. Antibiotic resistance of subgingival species during and after antibiotic therapy. J Clin Periodontol. 2002;29:724–35. 12390569 10.1034/j.1600-051x.2002.290809.x

[54] Seppala H, Klaukka T, Vuopio-Varkila J, Muotiala A, Helenius H, Lager K, et al The effect of changes in the consumption of macrolide antibiotics on erythromycin resistance in group A streptococci in Finland. Finnish Study Group for Antimicrobial Resistance. The New England journal of medicine. 1997;337:441–6. doi: 10.1056/NEJM199708143370701 9250845 10.1056/NEJM199708143370701

[55] Joshi JM. Tuberculosis chemotherapy in the 21 century: Back to the basics. Lung India. 2011;28:193–200. doi: 10.4103/0970-2113.83977 21886955 10.4103/0970-2113.83977 PMC3162758

[56] Milo G, Katchman EA, Paul M, Christiaens T, Baerheim A, Leibovici L. Duration of antibacterial treatment for uncomplicated urinary tract infection in women. Cochrane Database Syst Rev. 2005:CD004682 doi: 10.1002/14651858.CD004682.pub2 15846726 10.1002/14651858.CD004682.pub2 PMC12700497

[57] Dunbar LM, Wunderink RG, Habib MP, Smith LG, Tennenberg AM, Khashab MM, et al High-dose, short-course levofloxacin for community-acquired pneumonia: a new treatment paradigm. Clin Infect Dis. 2003;37:752–60. doi: 10.1086/377539 12955634 10.1086/377539

[58] Chastre J, Wolff M, Fagon JY, Chevret S, Thomas F, Wermert D, et al Comparison of 8 vs 15 days of antibiotic therapy for ventilator-associated pneumonia in adults: a randomized trial. Jama. 2003;290:2588–98. doi: 10.1001/jama.290.19.2588 14625336 10.1001/jama.290.19.2588

[59] de Boer WA, van Etten RJ, Schade RW, Ouwehand ME, Schneeberger PM, van Unnik AJ, et al One-day intensified lansoprazole-quadruple therapy for cure of Helicobacter pylori infection. Alimentary pharmacology & therapeutics. 1997;11:109–12. 10.1046/j.1365-2036.1997.121292000.x 9042982

[60] Green SM, Rothrock SG. Single-dose intramuscular ceftriaxone for acute otitis media in children. Pediatrics. 1993;91:23–30. 8416502

[61] Gooch WM 3rd, Blair E, Puopolo A, Paster RZ, Schwartz RH, Miller HC, et al Effectiveness of five days of therapy with cefuroxime axetil suspension for treatment of acute otitis media. The Pediatric infectious disease journal. 1996;15:157–64. 8822290 10.1097/00006454-199602000-00013

[62] Barnett ED, Teele DW, Klein JO, Cabral HJ, Kharasch SJ. Comparison of ceftriaxone and trimethoprim-sulfamethoxazole for acute otitis media. Greater Boston Otitis Media Study Group. Pediatrics. 1997;99:23–8. 8989332 10.1542/peds.99.1.23

[63] Schrag SJ, Pena C, Fernandez J, Sanchez J, Gomez V, Perez E, et al Effect of short-course, high-dose amoxicillin therapy on resistant pneumococcal carriage: a randomized trial. JAMA. 2001;286:49–56. 11434826 10.1001/jama.286.1.49

[64] Matuliene G, Pjetursson BE, Salvi GE, Schmidlin K, Bragger U, Zwahlen M, et al Influence of residual pockets on progression of periodontitis and tooth loss: results after 11 years of maintenance. J Clin Periodontol. 2008;35:685–95. doi: 10.1111/j.1600-051X.2008.01245.x 18549447 10.1111/j.1600-051X.2008.01245.x

[65] Armitage GC. Development of a classification system for periodontal diseases and conditions. Annals of periodontology / the American Academy of Periodontology. 1999;4:1–6. 10.1902/annals.1999.4.1.1 10863370

[66] O'Leary TJ, Drake RB, Naylor JE. The plaque control record. Journal of periodontology. 1972;43:38 doi: 10.1902/jop.1972.43.1.38 4500182 10.1902/jop.1972.43.1.38

[67] Ammenheuser MM, Hastings DA, Whorton EB Jr., Ward JB Jr. Frequencies of hprt mutant lymphocytes in smokers, non-smokers, and former smokers. Environmental and molecular mutagenesis. 1997;30:131–8. 9329637

[68] Ainamo J, Bay I. Problems and proposals for recording gingivitis and plaque. International dental journal. 1975;25:229–35. 1058834

[69] Lang NP, Tonetti MS. Periodontal risk assessment (PRA) for patients in supportive periodontal therapy (SPT). Oral Health Prev Dent. 2003;1:7–16. 15643744

[70] van Winkelhoff AJ, Winkel EG, Vandenbroucke-Grauls CM. On the dosage of antibiotics in clinical trials. J Clin Periodontol. 1999;26:764–6. 10589814 10.1034/j.1600-051x.1999.t01-10-261101.x

[71] Goodson JM, Haffajee AD, Socransky SS, Kent R, Teles R, Hasturk H, et al Control of periodontal infections: a randomized controlled trial I. The primary outcome attachment gain and pocket depth reduction at treated sites. J Clin Periodontol. 2012;39:526–36. doi: 10.1111/j.1600-051X.2012.01870.x 22512461 10.1111/j.1600-051X.2012.01870.x

[72] Katchman EA, Milo G, Paul M, Christiaens T, Baerheim A, Leibovici L. Three-day vs longer duration of antibiotic treatment for cystitis in women: systematic review and meta-analysis. Am J Med. 2005;118:1196–207. doi: 10.1016/j.amjmed.2005.02.005 16271900 10.1016/j.amjmed.2005.02.005

[73] Ardila CM, Granada MI, Guzman IC. Antibiotic resistance of subgingival species in chronic periodontitis patients. J Periodontal Res. 2010;45:557–63. doi: 10.1111/j.1600-0765.2010.01274.x 20546113 10.1111/j.1600-0765.2010.01274.x

[74] Jaramillo A, Arce RM, Herrera D, Betancourth M, Botero JE, Contreras A. Clinical and microbiological characterization of periodontal abscesses. J Clin Periodontol. 2005;32:1213–8. doi: 10.1111/j.1600-051X.2005.00839.x 16268997 10.1111/j.1600-051X.2005.00839.x

[75] Kulik EM, Lenkeit K, Chenaux S, Meyer J. Antimicrobial susceptibility of periodontopathogenic bacteria. J Antimicrob Chemother. 2008;61:1087–91. doi: 10.1093/jac/dkn079 18326855 10.1093/jac/dkn079

[76] Olsvik B, Hansen BF, Tenover FC, Olsen I. Tetracycline-resistant micro-organisms recovered from patients with refractory periodontal disease. J Clin Periodontol. 1995;22:391–6. 7601921 10.1111/j.1600-051x.1995.tb00166.x

[77] van Winkelhoff AJ, Herrera D, Oteo A, Sanz M. Antimicrobial profiles of periodontal pathogens isolated from periodontitis patients in The Netherlands and Spain. J Clin Periodontol. 2005;32:893–8. doi: 10.1111/j.1600-051X.2005.00782.x 15998275 10.1111/j.1600-051X.2005.00782.x

[78] van Winkelhoff AJ, Herrera Gonzales D, Winkel EG, Dellemijn-Kippuw N, Vandenbroucke-Grauls CM, Sanz M. Antimicrobial resistance in the subgingival microflora in patients with adult periodontitis. A comparison between The Netherlands and Spain. J Clin Periodontol. 2000;27:79–86. 10703651 10.1034/j.1600-051x.2000.027002079.x

[79] Walker CB. The acquisition of antibiotic resistance in the periodontal microflora. Periodontol 2000. 1996;10:79–88. 9567938 10.1111/j.1600-0757.1996.tb00069.x

[80] Herrera D, Contreras A, Gamonal J, Oteo A, Jaramillo A, Silva N, et al Subgingival microbial profiles in chronic periodontitis patients from Chile, Colombia and Spain. J Clin Periodontol. 2008;35:106–13. 10.1111/j.1600-051X.2007.01170.x 18081862

[81] Harks I, Koch R, Eickholz P, Hoffmann T, Kim TS, Kocher T, et al Is progression of periodontitis relevantly influenced by systemic antibiotics? A clinical randomized trial. J Clin Periodontol. 2015;42:832–42. doi: 10.1111/jcpe.12441 26250060 10.1111/jcpe.12441 PMC5054899

[82] van Winkelhoff AJ, Rodenburg JP, Goene RJ, Abbas F, Winkel EG, de Graaff J. Metronidazole plus amoxycillin in the treatment of Actinobacillus actinomycetemcomitans associated periodontitis. J Clin Periodontol. 1989;16:128–31. 2921374 10.1111/j.1600-051x.1989.tb01626.x

[83] Altamimi S, Khalil A, Khalaiwi KA, Milner R, Pusic MV, Al Othman MA. Short versus standard duration antibiotic therapy for acute streptococcal pharyngitis in children. Cochrane Database Syst Rev. 2009:CD004872 doi: 10.1002/14651858.CD004872.pub2 19160243 10.1002/14651858.CD004872.pub2

[84] Altamimi S, Khalil A, Khalaiwi KA, Milner RA, Pusic MV, Al Othman MA. Short-term late-generation antibiotics versus longer term penicillin for acute streptococcal pharyngitis in children. Cochrane Database Syst Rev. 2012:CD004872 doi: 10.1002/14651858.CD004872.pub3 22895944 10.1002/14651858.CD004872.pub3 PMC11984625

[85] Michael M, Hodson EM, Craig JC, Martin S, Moyer VA. Short versus standard duration oral antibiotic therapy for acute urinary tract infection in children. Cochrane Database Syst Rev. 2003:CD003966 doi: 10.1002/14651858.CD003966 12535494 10.1002/14651858.CD003966

[86] Hedrick TL, Evans HL, Smith RL, McElearney ST, Schulman AS, Chong TW, et al Can we define the ideal duration of antibiotic therapy? Surg Infect (Larchmt). 2006;7:419–32. 17083308 10.1089/sur.2006.7.419

[87] Sawyer RG, Claridge JA, Nathens AB, Rotstein OD, Duane TM, Evans HL, et al Trial of short-course antimicrobial therapy for intraabdominal infection. N Engl J Med. 2015;372:1996–2005. doi: 10.1056/NEJMoa1411162 25992746 10.1056/NEJMoa1411162 PMC4469182

[88] Lundgren D, Asklow B, Thorstensson H, Harefeldt AM. Success rates in periodontal treatment as related to choice of evaluation criteria. Presentation of an evaluation criteria staircase for cost-benefit use. J Clin Periodontol. 2001;28:23–30. 11142663 10.1034/j.1600-051x.2001.280104.x

[89] Matuliene G, Studer R, Lang NP, Schmidlin K, Pjetursson BE, Salvi GE, et al Significance of Periodontal Risk Assessment in the recurrence of periodontitis and tooth loss. J Clin Periodontol. 2010;37:191–9. doi: 10.1111/j.1600-051X.2009.01508.x 20041980 10.1111/j.1600-051X.2009.01508.x

[90] Van der Velden U, Varoufaki A, Hutter JW, Xu L, Timmerman MF, Van Winkelhoff AJ, et al Effect of smoking and periodontal treatment on the subgingival microflora. Journal of clinical periodontology. 2003;30:603–10. 12834497 10.1034/j.1600-051x.2003.00080.x

[91] Hughes FJ, Syed M, Koshy B, Bostanci N, McKay IJ, Curtis MA, et al Prognostic factors in the treatment of generalized aggressive periodontitis: II. Effects of smoking on initial outcome. Journal of clinical periodontology. 2006;33:671–6. doi: 10.1111/j.1600-051X.2006.00965.x 16856898 10.1111/j.1600-051X.2006.00965.x

[92] Faveri M, Rebello A, de Oliveira Dias R, Borges-Junior I, Duarte PM, Figueiredo LC, et al Clinical and microbiologic effects of adjunctive metronidazole plus amoxicillin in the treatment of generalized chronic periodontitis: smokers versus non-smokers. J Periodontol. 2014;85:581–91. doi: 10.1902/jop.2013.130278 23826648 10.1902/jop.2013.130278

