# Supplementary material for: One year results of a randomized controlled clinical study evaluating the effects of non-surgical periodontal therapy of chronic periodontitis in conjunction with three or seven days systemic administration of amoxicillin/metronidazole
Source: PLoS One. 2017 Jun 29;12(6):e0179592. doi: 10.1371/journal.pone.0179592 (PMC5491014; doi:10.1371/journal.pone.0179592)
Supplement: S1 Table — ITT = intention to treat, PD = pocket depth, CAL = clinical attachment level, BOP = bleeding on probing; FMPS = full-mouth plaque score after O’Leary [66], m: months, base: baseline. s statistically significant p values. (DOCX) [file pone.0179592.s001.docx]

# Supporting information

**S1 Table ITT analyses: mean values and group comparisons (ANOVA adjusted for smoking, ANCOVA adjusted for baseline values and smoking for 12 month variables), and their changes (Δ) between baseline and 12 months (mean±SD)**

| **Variables** | | **Group A** | | **Group B** | | **Group C** | | **Group comparisons** | | | | | | **Smoker - Non-smoker** | |
| --- | --- | --- | --- | --- | --- | --- | --- | --- | --- | --- | --- | --- | --- | --- | --- |
| **Baseline** | | **(SRP+Placebo)N=30** | | **(SRP+AB 3d) N=30** | | **(SRP+AB 7d) N=31** | | ***p* value** | | ***p* value** | | ***p* value** | |  | |
| **12m** | | **N=27** | | **N=26** | | **N=27** | | **A-B** | | **A-C** | | **B-C** | | ***P value*** | |
| **PD (mm)** | |  | |  | |  | |  | |  | |  | |  | |
| Baseline | | 5.43±0.60 | | 5.46±0.50 | | 5.74±0.57 | | 1.000 | | 0.127 | | 0.211 | | 0.035^s^ | |
| 12 m | | 3.33±0.46 | | 2.89±0.47 | | 3.00±0.54 | | 0.003^s^ | | 0.030 ^s^ | | 1.000 | | 0.015 ^s^ | |
| **∆ baseline- 12m** | | 2.07±0.69 | | 2.57±0.65 | | 2.71±0.82 | | 0.033^s^ | | 0.005^s^ | | 1.000 | | 0.004^s^ | |
| **CAL (mm)** | |  | |  | |  | |  | |  | |  | |  | |
| Baseline | | 5.89±1.24 | | 5.84±1.19 | | 6.06±1.15 | | 1.000 | | 1.000 | | 1.000 | | 0.713 | |
| 12 m | | 4.69±1.15 | | 4.29±1.24 | | 4.29±1.16 | | 0.022 ^s^ | | 0.005 ^s^ | | 1.000 | | 0.002^s^ | |
| **∆ baseline- 12m** | | 1.19±0.68 | | 1.62±0.65 | | 1.73±0.54 | | 0.035 ^s^ | | 0.006^s^ | | 1.000 | | 0.009^s^ | |
| **BoP (%)** | |  | |  | |  | |  | |  | |  | |  | |
| Baseline | | 64.93±28.66 | | 64.06±34.33 | | 71.27±26.55 | | 0.795 | | 1.000 | | 1.000 | | 0.762 | |
| 12 m | | 13.21±9.90 ^s^ | | 10.21±6.87 ^s^ | | 11.23±9.54 ^s^ | | 0.669 | | 1.000 | | 1.000 | | 0.553 | |
| **∆ baseline- 12m** | | 53.75±25.38 | | 51.27±35.92 | | 57.52±29.65 | | 1,000 | | 1,000 | | 1.000 | | 0.794 | |
| **FMPS (%)** | |  | |  | |  | |  | |  | |  | |  | |
| Baseline | | 19.86±6.11 | | 17.29±6.04 | | 19.51±5.72 | | 1.000 | | 0.256 | | 1.000 | | 0.111 | |
| 12 m | | 21.54±12.76 | | 33.89±23.35^s^ | | 30.31±19.76^s^ | | 0.071 | | 0.332 | | 1.000 | | 0.032^s^ | |
| **∆ baseline- 12m** | | -0.94±12.23 | | -16.08±22.92 | | -10.98±19.99 | | 0.020^s^ | | 0.227 | | 0.914 | | 0.120 | |
| **PD 4-6 mm (mm)** | |  | |  | |  | |  | |  | |  | |  | |
| Baseline | | 4.88±0.22 | | 4.90±0.25 | | 4.92±0.22 | | 1.000 | | 1.000 | | 1.000 | | 0.489 | |
| 12 m | | 3.09±0.41 | | 2.72±0.40 | | 2.78±0.46 | | 0.004^s^ | | 0.031^s^ | | 1.000 | | 0.003^s^ | |
| **∆ baseline- 12m** | | 1.78±0.47 | | 2.19±0.50 | | 2.17±0.48 | | 0.007^s^ | | 0.014^s^ | | 1.000 | | 0.005^s^ | |
| **CAL of PD 4-6 mm (mm)** | |  | |  | |  | |  | |  | |  | |  | |
| Baseline | | 5.47±1.10 | | 5.32±1.14 | | 5.32±0.98 | | 1.000 | | 1.000 | | 1.000 | | 0.640 | |
| 12 m | | 4.45±1.04 | | 4.06±1.21 | | 3.92±1.11 | | 0.072 | | 0.012^s^ | | 1.000 | | 0.005 ^s^ | |
| **∆ baseline- 12m** | | 1.03±0.61 | | 1.34±0.58 | | 1.44±0.42 | | 0.104 | | 0.021^s^ | | 1.000 | | 0.011^s^ | |
| **PD >7 mm (mm)** | |  | |  | |  | |  | |  | |  | |  | |
| Baseline | | 7.51±0.55 | | 7.63±0.62 | | 7.86±0.84 | | 1.000 | | 0.519 | | 0.645 | | 0.797 | |
| 12m | | 4.48±1.12 | | 3.58±0.92 | | 3.70±0.93 | | 0.004 ^s^ | | 0.021 ^s^ | | 1.000 | | 0.022 ^s^ | |
| **∆ baseline- 12m** | | 3.09±1.30 | | 4.06±0.97 | | 4.08±1.44 | | 0.017^s^ | | 0.016 ^s^ | | 1.000 | | 0.077 | |
| **CAL of PD >7 mm (mm)** | |  | |  | |  | |  | |  | |  | |  | |
| Baseline | | 7.67±1.00 | | 7.97±1.49 | | 7.88±1.43 | | 1.000 | | 1.000 | | 1.000 | | 0.356 | |
| 12 m | | 5.67±1.25 | | 5.35±1.65 | | 5.26±1.46 | | 0.089 | | 0.434 | | 1.000 | | 0.014^s^ | |
| **∆ baseline- 12m** | | 1.98±1.21 | | 2.68±1.12 | | 2.42±0.79 | | 0.051 | | 0.456 | | 0.936 | | 0.039 ^s^ | |
| **No. PD≥6mm** | |  | |  | |  | |  | |  | |  | |  | |
| Baseline | | 25.57±16.51 | | 30.70±14.94 | | 35.48±16.66 | | 0.654 | | 0.066 | | 0.845 | | 0.238 | |
| 12 m | | 5.46±4.44 | | 1.68±2.21 | | 4.22±5.30 | | 0.002^s^ | | 0.463 | | 0.127 | | 0.037^s^ | |
| **∆ baseline- 12m** | | 20.28±16.07 | | 28.04±14.78 | | 30.85±17.09 | | 0.241 | | 0.061 | | 1.000 | | 0.114 | |
| **No. PD≥5mm** | |  | |  | |  | |  | |  | |  | |  | |
| Baseline | | 42.36±19.46 | | 48.30±19.85 | | 52.96±19.52 | | 0.741 | | 0.124 | | 1.000 | | 0.726 | |
| 12 m | | 11.36±9.02 | | 4.64±5.29 | | 8.11±8.65 | | 0.002 ^s^ | | 0.206 | | 0.320 | | 0.001 ^s^ | |
| **∆ baseline- 12m** | | 31.07±19.21 | | 41.84±18.36 | | 44.55±20.55 | | 0.138 | | 0.043^s^ | | 1.000 | | 0.134 | |
| **No. PD=4 mmBOP+ and PD≥5mm** | |  | |  | |  | |  | |  | |  | |  | |
| Baseline | | 56.40±23.46 | | 62.83±22.31 | | 67.32±22.47 | | 0.838 | | 0.202 | | 1.000 | | 0.906 | |
| 12 m | | 13.53±10.94 | | 6.36±6.13 | | 10.81±10.59 | | 0.006 ^s^ | | 0.491 | | 0.224 | | 0.001^s^ | |
| **∆ baseline- 12m** | | 42.89±21.37 | | 54.28±21.22 | | 54.74±23.23 | | 0.184 | | 0.173 | | 1.000 | | 0.121 | |

ITT= intention to treat, PD= pocket depth, CAL=clinical attachment level, BOP=bleeding on probing; FMPS= full-mouth plaque score after O’Leary ([O'Leary et al. 1972](#_ENREF_58)), m: months, base: baseline.

^s^  statistically significant *p* values.
