# Supplementary material for: One year results of a randomized controlled clinical study evaluating the effects of non-surgical periodontal therapy of chronic periodontitis in conjunction with three or seven days systemic administration of amoxicillin/metronidazole
Source: PLoS One. 2017 Jun 29;12(6):e0179592. doi: 10.1371/journal.pone.0179592 (PMC5491014; doi:10.1371/journal.pone.0179592)
Supplement: S1 Text — (PDF) [file pone.0179592.s003.pdf]

Study protocol for the ethical committee
University of Cluj-Napoca

Titelof the study:

Non-surgical periodontal therapy in conjunction with two different durations of Amoxicillin and Metronidazole administration in patients with severe chronic periodontitis

Centers:

Clinic for Periodontology,
University Bern, Switzerland
Clinic director:
Prof. Dr. med. dent.Anton Sculean, Dr. h.c., M.S.
PD. Dr. med. dent.SigrunEick
Freiburgstrasse7
3010 Bern, Switzerland

Clinic for Prosthetic Dentistry,
University „IuliuHatieganu“, Cluj-Napoca, Romania
Study coordinator: Dr. Raluca Cosgarea
Str. Clinicilor 32
3400 Cluj-Napoca, Romania

Estimated time interval forthe study:

2012-2014

Introduction

Periodontitis is an inflammatory disease initiated by bacteria residing in biofilms at and below the gingival margins, affecting the tissues surrounding the teeth; it involves progressive loss of the alveolar bone and, if left untreated, may lead to tooth-loss. Periodontal therapy includes the removal of bacterial deposits from the tooth structures through mechanical cleaning ("scaling and root planing", SRP). However, it has been shown that SRP cannot always be performed effectively enough in pockets deeper than 5 mm [Badersten et al. 1987, Caffesse et al. 1986]. Therefore, periodontal treatment is performed in two stages: a non-surgical subgingival debridement and a surgical corrective therapy [Heitz-Mayfield et al. 2002, van der Weijden et al. 2002].

However, periodontopathogenic bacteria cannot be completely eliminated through mechanical debridement alone. Bacteria can be found in dentin tubules, lacunae, concavities and, by invading soft tissues, are unreachable when using mechanical instruments. Several studies have proved the resistance of *Aggregatibacter actinomycetemcomitans (A.a.)* to mechanical therapy alone and associated progressive tissue destruction with the persistance of *A.a., Porphyromonas gingivalis (P.g.)* and some other bacterial species [Mombelli et al. 1994, Mombelli et al 2000, Renvert et al. 1990]. Better clinical outcomes were observed when the periodontopathogenic bacteria were undetectable [Dahlen et al. 1996, Grossi et al. 1994, Haffajee&Sokransky 1994].

Given this limitations, mechanical debridement has been completed with the adjunctive use of antibacterial agents (antibiotics and antiseptics). Because of the complex structure of the bacterial biofilm [Marsch et al. 2005], biofilm bacteria show a reduced susceptibility to antimicrobials as compared to free floating bacteria. Therefore, the use of adjunctive antibiotics has been used concomitant to subgingival debridement, when the disruption of the biofilm is possible.

A large scale of antibiotics have been investigated as possible adjuncts to SRP: amoxicillin (with and without clavulanic acid), metronidazole, clindamycin, doxycycline, azithromycin, moxifloxacin, tetracycline, spiramycin and combinations of these [Slots et al. 2004, Guentsch et al 2008, Griffiths et al. 2011, Flemmig et al. 2011, Herrera et al. 2008, Heitz-Mayfield et al. 2009]. The combination of Amoxicillin (AMX) and Metronidazole (MET) has mostly been investigated and was shown to suppress *A.a*ctinomycetemcomitans and other periodontopathogenic bacteria from the periodontal compromised sites [van Winkelhoff et al 1991, Winkel et al. 2001, Rooney et al. 2002, Matarazzo et al. 2008, Lopez et al. 1998, Guerrero et al. 2005]. Also, better clinical results measured in reduction of probing pocket depth (PD) and clinical attachment level (CAL) gain as compared to placebo were obtained in several clinical studies [Cionca et al. 2010, 2009, Ribeiro et al. 2009, Griffiths et al. 2011, Heller et al. 2011, Mestnik et al. 2010, Varela et al. 2011, Yek et al. 2010, Silva et al. 2011]. Furthermore, a statistically significant reduction of the periodontopathogens as well as cytokines could be reported in several studies [Cionca et al. 2010, 2009, Ribeiro et al. 2009, Heller et al. 2011, Mestnik et al. 2010, Yek et al. 2010, Silva et al. 2011, Oliveira et al. 2011, Engebretson et al. 2002, Rosalem et al. 2011, Goutoudi et al. 2004].

However, the dosage and duration of the prescribed antibiotics varies in the literature. Several studies evaluated the effect of 375 mg AMX and 250/500 mg MET 3 t.i.d. prescribed for 7-8 d as an adjunctive to SRP [Cionca et al. 2010, 2009, Flemmig et al. 1998, Mombelli et al. 2005, Ehmke et al. 2005, Ribeiro et al. 2009]; in other studies both antibiotics were given in a dosage of 500 mg 3 t.i.d. for 7 d[Yek et al 2010, Griffiths et al 2011, Guerrero et a. 2005], or 500 mg AMX and 400/250 mg MET 3.i.d. for 10 to 14 d [Heller et al. 2011, Silva et al. 2011, Matarazzo et al. 2008, Mestnik et al. 2010, Rodriguez et al. 2011]. Whatever dosage and duration is prescribed, it is important that antibiotics should be taken in sufficient dose for adequate duration.

Aim

The aim of the present study is to evaluate the clinical, microbiological and immunological outcomes following non-surgical periodontal therapy (performed within 24 hours) in conjunction with 2 durations of adjunctive Amoxicillin (AMX) and Metronidazole (MET) in patients with advanced chronic periodontitis (ChP).

Objectives

Primary objective:

- To evaluate Clinical Attachment Level (CAL) changes after fm SRP (performed within 24 hours) in conjunction with 2 different dosages and durations (i.e. 3 or 7 days) of adjunctive Amoxicillin (AMX) and Metronidazole (MET) after 3, 6 and 12 months.

Secondary objectives:

- Evaluation of the changes in probing depth (PPD) reduction and bleeding on probing (BOP).
- To explore the effect on the subgingival bacterial load and microbial colonisation.
- To determine the effects on the GCF cytokines (IL-1β, IL-10, IL-8, MMP-8).
- Postoperative morbidity and patient-centered outcomes.
- Adverse reactions.

Patients:

102 patients with severe chronic periodontitis (with at least 1 pocket per quadrant of a depth of ≥ 7 mm) will be recruited at the Clinic for Prosthetic Dentistry University “Iuliu Hatieganu” Cluj-Napoca, Romania.

Inclusion criteria:

Patients

- Minimum age 18.

- ≥12 natural teeth present.
- clinical and radiographic signs of severe (PPD ≥6 mm) chronic periodontitis.
- good level of oral hygiene [plaque control record (PCR) after O'Leary 1972 ≤25%].
- systemically healthy: no history of diseases that may influence the severity or progression of the periodontal disease (Down Syndrome, HIV, Diabetes Mellitus type 1 and 2), post-iradiation in the head and neck area, infectious diseases or heart diseases that need a prophilactic antibiosis before dental treatments, liver diseases.
- informed written consent.

Exclusion criteria:

- subgingival debridement within the previous 12 months.
- systemic or local use of antibiotics within the preceding 3 months.
- Medication that may interact with AMX or MET (e.g., coumarin derivates, containing alcohol derivates, 5-fluor-uracyl/ disulfiram derivates, amprenavir oral solutions, lopinavir/ritonavir oral solution).
- Medication that may influence the periodontium: Ciclosporin A, compounds of Phenytoin, calcium channel blockers (Nifedipine, Verapamile, Amlodipine, Diltiazeme).
- Pregnancy or lactation.
- Patients who don't match the inclusion criteria.

Studytype:

Prospective, randomized, clinical trial.

Study design:

Clinical examination

One blinded and calibrated investigator will perform all clinical examinations. The following data will be assessed at baseline (before fm SRP), 3, 6 and 12 months after non-surgical therapy:

- Medical history.
- Smoking history; patients smoking >10 cigarettes per day will be considered smokers [Tonetti et al. 1995] and patients who stopped smoking 5 years before the beginning of the study will be considered former smokers [Ramseier& Lang 1999].
- Dental status.
- Periodontal status including probing pocket depth (PPD), clinical vertical attachment level (CAL) at 6 sites per tooth with a periodontal probe (PCPUNC 15; Hu Friedy®, Chicago, IL, USA) at the nearest 0.5mm, furcation involvement [Hamp et al. 1975] with a Nabers probe(PQ2N, Hu Friedy®, Chicago, IL, USA) and mobility assessment. As a

reference point for the CAL-V measurements the cement-enamel junction (CEJ) will be used. If the CEJ is destroyed by a restoration (filling or crown) the margin of the restoration will be considered as a reference point.

- Bleeding on probing (BOP).
- Suppuration on probing (SOP).
- Gingival bleeding index (GBI according to Ainamo& Bay 1975) as percentage of sites with the presence of bleeding as determined at four sites (mesial, buccal, distal and oral) per toothafter running the probe along the soft tissue margin.
- Plaque control record (PCR according to O'Leary 1972) as percentage of sites with the presence of plaque as determined at four sites (mesial, buccal, distal and oral) per tooth after staining with disclosing solution.
- Panoramic radiograph prior to fm SRP if none was made within the previous12 months.
- Microbial and GCF samples will be obtained at baseline from the deepest site in each quadrant.

Intra-examiner reproducibility

The examiner is calibrated by measuring PPD, CAL and FI in five patients two times, 48h apart. Positive calibration is considered when both measurements are within one millimetre more than 90% of the times.

GBI (GingivalBleeding Index) according to Ainamo and Bay [1975]

Thisindexrenders in percentage the gingivalbleedingaroundallteeth. Everytoothisdevided in 4sectors: mesial, distal, buccal and oral. 15s afterrunning a periodontal probe (PCP UNC 12 PT, HuFriedy, Chicago, IL, USA) over the gingivalmargin, the percentage of bleedingpointswill be calculated.

PCR (Plaque Control Record) according to O´Leary [1972]

This index renders in percentage the dental plaque around the whole dentition. Everytoothisdevided in 4sectors: mesial, distal, buccal and oral. After plaque staining with disclosing solution, all tooth sectors marked with dental plaque in the gingival crown area will be counted according to dichotomic decision. The plaque index will be calculated in percentages of the marked over the total surfaces.

GCF sampling

After relatively drying up the region of interest with cotton rolls, supragingival dental plaque will be removed. A standard sterile paper strip (Periopaper, Oraflow Inc., Smithtown, NY,

USA) will beheld for 30s at the gingival crevice and stored at -70° untilcollection of allGCFsamples.

Microbial sampling

After relatively drying up the region of interest with cotton rolls, supragingival dental plaque will be removed. Sterile paper cones will be inserted up to the bottom of the pockets for 20s and stored at -20° untilcollection of allmicrobiologicalsamples.

Cytokine analysis

Following host-derived biomarkers will be determined with ELISA: IL-1β, IL-10, IL-8, MMP-8.

Microbiology

Following periodontal pathogens will be determined by real-time PCR method:

Aggregatibacter actinomycetemcomitans, Porphyromonas gingivalis, Tannerella forsythia, and *Treponema denticola.*

Randomisation

According to a computer-generated randomization list, patients will be assigned to one of the following treatment groups:

Test 1 (T1): SRP plus AMX (500 mg t.i.d., for 7 days) and MET (500 mg t.i.d. for 7 days).

Test 2 (T2): SRP plus AMX (500 mg t.i.d., for 3 days) and MET (500 mg t.i.d. for 3 days).

Control (C): SRP alone

The randomization list will be concealed to the patient, clinical examiner, therapist and statistician.

Clinical Procedure

Two independent clinicians will perform all the procedures involving contact to the patients: one will enrol and assess all the clinical parameters, as well as take the GCF and microbiological samples, while the other will perform the SRP procedure after the patients will have reached the adequate plaque control.

Within 24h, SRP will be performed at all periodontally diseased teeth (PD≥ 4 mm) under local anaesthesia. Ultrasonic instruments (Cavitron, Kavo, Biberach, Germany) will be used first, followed by Gracey curettes (Hu-Friedy, Chicago, IL, USA) and again by ultrasonic instruments. All treated pockets will be irrigated with 1% chlorhexidine gel.

Postoperative care

Postoperative care will consist of mouth rinses with 0.12% chlorhexidinedigluconate solution for 2 min twice a day for 2 weeks and tooth brushing with 1% chlorhexidine gel. Patients will receive the antibiotics according to the randomisation list (see subchapter 'Randomisation')..

Recall appointments will be scheduled 2 weeks after fm SRP, when CHX staining will be removed. At two weeks, first GCF samples will be obtained. Then recalls will be scheduled 3, 6 and 12 months after fm SRP when clinical parameters, GCF and microbial samples will be taken, as well as supragingival scaling and polishing will be performed.

Statistical design

The statistical analysis will be performed using the commercially available software program SPSS for Windows Version 12.0). Mean values and standard deviations will be calculated for each clinical variable. Median and range will be. Parameter free tests will be used.

The primary outcome variable will be considered the change of PD.

CAL, recession change, BOP, microbial loads and the cytokine levels will be considered as secondary endpoints.

Compliance and adverse effects

Patients will be asked to bring the medication packs back so as to check the compliance. They will answer a questionnaire about self-perceived side-effects.

Data documentation:

The acquired data will be documented on CRFs. The coordinator of the study will assure the safe deposit of the patient data.

Ethical aspects:

The study will be performed in accordance with the Declaration of Helsinki 1975, as revised in 2000.

All participants will have signed the informed written consent prior to the study begin.

The study protocol will be submitted to the ethics committee of the University of Cluj-Napoca. Patient recruitment will not begin without a prior positive consent from the ethics committee.

All negative occurrences will be thoroughly documented.

Signatures

Study coordinator:

Prof.Dr. med. dent. Anton Sculean, Dr.h.c., M.S.

Dr. med dent. Raluca Cosgarea

PD. Dr. med. dent. Sigrun Eick (for the laboratory analysis)

References

1. Ainamo, J., Bay, I. (1975) Problems and proposals for recording gingivitis and plaque. Int Dent J 25, 229-235.

2. Badersten A, Nilve´us R, Egelberg J. Effect of nonsurgical periodontal therapy (VIII). Probing attachment changes related to clinical characteristics. J ClinPeriodontol 1987: 14: 425–432.

3. Caffesse RG, Sweeney PL, Smith BA. Scaling and root planing with and without periodontal flap surgery. J ClinPeriodontol 1986: 13: 205–210.

4. Cionca N, Giannopoulou C, Ugolotti G, Mombelli A. Amoxicillin and metronidazole as an adjunct to full-mouth scaling and root planing of chronic periodontitis. J Periodontol 2009: 80: 364–371.

5. Cionca N, Giannopoulou C, Ugolotti G, Mombelli A. Microbiological testing and outcomes of full-mouth scaling and root planing with or without amoxicillin ⁄ metronidazole in chronic periodontitis. J Periodontol 2010: 81: 15–23.

6. Dahlen G, Wikström M, Renvert S. Treatment of periodontal disease based on microbiological diagnosis. A 5–year follow-up on individual patterns. J Periodontol 1996: 67: 879–887.

7. Ehmke, B., Moter, A., Beikler, T., Milian, E. &Flemmig, T. F. Adjunctive antimicrobial therapy of periodontitis: long-termeffects on disease progression and oral colonization. Journal of Periodontology 2005: 76, 749–759.

8. Engebretson, S. P., Grbic, J. T., Singer, R. &Lamster, I. B. GCF IL-1beta profiles in periodontal disease. Journal of Clinical Periodontology 2002: 29, 48–53.

9. Flemmig TF, Petersilka G, Völp A, Gravemeier M, Yillz M, Mross D, Prior K, Zamamoto J, Beikler T. Efficacy and safety of adjunctive local moxifloxacin delivery in the treatment of periodontitis. JPeriodontol 2011: 82: 96-105.

10. Goutoudi, P., Diza, E. &Arvanitidou, M. Effect of periodontal therapy on crevicular fluid interleukin-1beta and interleukin-10 levels inchronic periodontitis. Journal of Dentistry 2004: 32, 511–520.

11. Griffiths GS, Ayob R, Guerrero A, Nibali L, Suvan J, Moles DR, Tonetti MS. Amoxicillin and metronidazole as an adjunctive treatment in generalized aggressive periodontitis at initial therapz or re-treatment: a randomized controlled clinical trial. J ClinPeriodontol 2011: 38: 43-49.

12. Grossi SG, Zambon JJ, Ho AW, Koch G, Dunford RG, Machtei EE, Norderyd OM, Genco RJ. Assessment of riskfor periodontal disease. I. Risk indicators for attachment loss. J Periodontol 1994: 65: 260–267.

13. Guentsch A, Jentsch H, Pfister W, Hoffmann T, Eick S. Moxifloxacin as anadjunctive antibiotic in the treatment of severe chronic periodontitis. JPeriodontol. 2008: 79:1894-903.

14. Guerrero A, Griffiths GS, Nibali L, Suvan J, Moles DR, Laurell L, Tonetti MS. Adjunctive benefits of systemic amoxicillin and metronidazole in non-surgical treatment of generalized aggressive periodontitis: a randomized placebo-controlled clinical trial. J ClinPeriodontol 2005: 32: 1096–1107.

15. Haffajee AD, Socransky SS. Microbial etiological agents of destructive periodontal diseases. Periodontol 2000 1994: 5: 78–111.

16. Hamp, S.E., Nymann, S., Lindhe, J. Periodontal treatment of multirooted teeth. Results after 5 years. J ClinPeriodontol1975: 2, 126-135.

17. Heitz-Mayfield LJ, Trombelli L, Heitz F, Needleman I, Moles D. A systematic review of the effect of surgical debridement vs non-surgical debridement for the treatment of chronic periodontitis. J ClinPeriodontol 2002: 29(Suppl. 3): 92–102.

18. Heitz-Mayfield LJ. Systemic antibiotics in periodontal therapy. Austr Dent J 2009: 54 (Suppl 1): S96-S101.

19. Heller D, Varela VM, e Silva-Senem MX, Torres MCB, Feres-Filho EJ, Colombo APV: Impact of systemic antimicrobials combined to anti-infective mechanica debridement on the microbiota of generalized aggressive periodontitis: a 6-month RCT. J ClinPeriodontol 2011: 38: 355-364.

20. Herrera D, Alonso B, Leon R, Roldan S, Sanz M. Antimicrobial therapy in periodontitis: the use of systemic antimicrobials against the subgingival biofilm. J ClinPeriodontol 2008: 35 (Suppl 8): 45-66.

21. Lopez N, Gamonal J. Effects of metronidazole plus amoxicillin in progressive untreated adult periodontitis: results of a single one-week course after 2 and 4 months. J Periodontol 1998: 69: 1291–1298.

22. Marsh PD. Dental plaque: biological significance of a biofilm and community life-style. J ClinPeriodontol 2005;32(Suppl 6):7–15.

23. Matarazzo F, Figueiredo LC, Cruz SEB, Faveri M, Feres M. Clinical and microbiological benefits of systemic metronidazole and amoxicillin in the treatment of smokers with chronic pperiodontiti: a randomized placebo-controlled study. J ClinPeriodontol: 35: 885-896.

24. Mestnik MJ, Feres M, Figueriredo LC, Duarte PM, Lira EAG, Faveri M. Short-term benefits of the adjunctive use of metronidazole plus amoxixillin in the microbial profile and in clinical parameters of subjects with generalized aggressive periodontitis. J Clin periodontal 2010: 37: 353-365.

25. Mombelli A, Gmu¨ r R, Gobbi C, Lang NP. Actinobacillus actinomycetemcomitans in adult periodontitis. I. Topographic distribution before and after treatment. J Periodontol 1994: 65: 820–826.

26. Mombelli A, Schmid B, Rutar A, Lang NP. Persistence patterns of Porphyromonasgingivalis, Prevotellaintermedia⁄ nigrescens, and Actinobacillusactinomycetemcomitans after mechanical therapy of periodontal disease. J Periodontol 2000: 71: 14–21.

27. Mombelli, A. Antimicrobial profiles of periodontal pathogens and systemic antimicrobial therapy. Journal of Clinical Periodontology 2005: 32, 891–892.

28. O'Leary, T.J., Drake, R.B., Naylor, J.E. The plaque control record. J Periodontol1972: 43, 38.

29. Oliveira APL, Faveri M, Gursky L, Mestnik MJ, Feres M, Haffajee AD, Socransky SS, Teles RP. Effects of periodontal therapy on GCF cytokines in generalized aggressive periodontitis. J ClinPeriodontolepub ahead of print. doi: 10.1111/j.1600-051X.2011.01817.x.

30. Renvert S, Wikstro¨m M, Dahle´n G, Slots J, Egelberg J. Effect of root debridement on the elimination of Actinobacillusactinomycetemcomitans and Bacteroidesgingivalis from periodontal pockets. J ClinPeriodontol 1990: 17: 345–350.

31. Rooney J, Wade WG, Sprague SV, Newcombe RG, Addy M. Adjunctive effects to non-surgical therapy of systemic metronidazole and amoxycillin alone and combined. A placebo controlled study. J ClinPeriodontol 2002: 29: 342– 350.

32. Rosalem, W., Rescala, B., Teles, R. P., Fischer, R. G., Gustafsson, A. &Figueredo, C. Effect of non-surgical treatment on chronic and aggressive periodontitis: clinical, immunological and microbiological findings. Journal of Periodontology 2011: 82, 979–989.

33. Ribeiro Del Peloso E, Bittencourt S, Zanin IC, BoviAmbrosano GM, Sallum EA, Nociti FH, Goncalves RB, Casati MZ. Full-mouth ultrasonic debridement associated with amoxicillin and metronidazole in the treatment of severechronic periodontitis. J Periodontol 2009: 80: 1254–1264.

34. Rodrigues AS, Lourencao DS, Neto LGL, Pannuti CM, Hirata RDC, Hirata MH, Lotufo RFM, Micheli G. Clinical and microbiological evaluation, by real-time PCR, of non-surgical treatment of aggressive periodontitis associated with amoxicillin and metronidazole. J Periodontol 2011, epubeahead of print DOI: 10.1902/jop.2011.110333.

35. Silva MP, Feres M, Sirotto TAO, Soares GMS, Mendes JAV, Faveri M, Figueiredo LC. Clinical and microbiological benefits of metronidazole alone or with amoxicillin as adjuncts in the treatment of chronic periodontitis: a randomized placebo-controlled clinical trial. J ClinPeriodontol 2011: 38: 828-837.

36. Slots J, Greenwell H, Fiorellini J, Giannobile W, Offenbacher S, Salkin L. Systemic antibiotics in periodontics. J Periodontol 2004: 75: 1553–1565.

37. van der Weijden GA, Timmerman FA. A systematic review on the clinical efficacy of subgingival debridement in the treatment of chronic periodontitis. J ClinPeriodontol 2002: 29(Suppl. 3): 55–71.

38. vanWinkelhoff AJ, Abbas F, Pavicic MJAMP, de Graaff J. Chronic conjunctivitis caused by oral anaerobes and effectively treated with systemic metronidazole plus amoxicillin. J ClinMicrobiol 1991: 29: 723–725.

39. Varela VM, Heller D, Silva-Senem MX, Torres MCMB; Colombo APVC, Feres-Filho EJ. Systemic antimicrobials adjunctive to a repeated mechanical and antiseptic therapz for aggressive periodontitis: a 6-month randomized controlled trial. J Periodontol 2011: 82: 1121-1130.

40. Winkel EG, van Winkelhoff AJ, Timmerman MF, van der Velden U, Van der Weijden GA. Amoxicillin plus metronidazole in the treatment of adult periodontitis patients. A double-blind placebo-controlled study. J ClinPeriodontol 2001: 28: 296–305.

41. Yek EC, Cintan S, Topcuoglu N, Kulekci G, Issever H, Kantarci A. Efficacy of amoxicillin and metronidazole combination for the management of generalized aggressive periodontitits. J Periodontol 2010: 81: 964-974.
