# Supplementary material for: One year results of a randomized controlled clinical study evaluating the effects of non-surgical periodontal therapy of chronic periodontitis in conjunction with three or seven days systemic administration of amoxicillin/metronidazole
Source: PLoS One. 2017 Jun 29;12(6):e0179592. doi: 10.1371/journal.pone.0179592 (PMC5491014; doi:10.1371/journal.pone.0179592)
Supplement: S2 Text — (PDF) [file pone.0179592.s004.pdf]

Protocolul studiului pentru depunerea la Comisia Etică a UMF „Iuliu Hatieganu“ Cluj-Napoca

09.01.2012

Titlul studiului:

Tratamentul parodontal nechirurgical cu administrare de Amoxicilina și Metronidazol pentru doua perioade de administrare diferită la pacientii cu parodontita cronică severă

Centre participante la studiu:

Clinica de parodontologie
Universitatea Berna, Elvetia
Director: Prof. Dr. med. Dent. Anton Sculean, Dr. h.c., M.S.
PD Dr. med dent. Sigrun Eick
Freiburgstr. 7, 3010 Berna, Elvetia

Clinica de protetica Dentara
Universitatea de Medicina si Farmacie “Iuliu Hatieganu”, Cluj-Napoca
Coordonator studiu: Sef lucrari Dr. Raluca Cosgarea
Str. Clinicilor Nr. 32, Cluj-Napoca
E-Mail: raluca.cosgarea@umfcluj.ro
Tel: 0751-638904

Timp estimat pentru desfasurarea studiului:

2012-2014

Introducere

Parodontita reprezintă o boală inflamatorie iniţiată de biofilmul bacterian şi care afectează ţesuturile ce înconjoară dinţii; boala implică resorbţie osoasă progresivă care, daca rămâne netratată poate sa ducă la pierderea dinţilor. Tratamentul parodontal implică îndepărtarea filmului bacterian de pe structura dintelui prin curățare mecanică (“scaling and root planing”, SRP). În schimb, a fost demonstrat ca SRP nu este destul de eficient în pungi parodontale (PPT) mai adânci de 5 mm [Badersten et al. 1987, Caffesse et al. 1986]. De aceea tratamentul parodontal este realizat intr-o etapă nechirurgicală și una chirurgicală corectivă [Heitz-Mayfield et al. 2002, van der Weijden et al. 2002].

Bacteriile parodontopatogene nu pot fi eliminate complet prin SRP. Multe studii au demonstrat persistența unor bacterii parodontopatogene (aggregatibacter actinomycetemcomitans, porphyromonas gingivalis, e.t.c.) după terapia mecanică și progresia distrucției parodontale în aceste situații [Mombelli et al. 1994, Mombelli et al 2000, Renvert et al. 1990].

Datorită acestor limitări, surfasajul mecanic radicular a fost completat de folosirea adjuvanta a antibioticelor. O scala largă de antibiotice au fost investigate în scopul folosirii ca si terapie adjuvanta la SRP: amoxicilina cu si fara acid clavulanic, metronidazol, clindamicina, doxiciclina, azitromicina, moxifloxacina, tetraciclina, spiramicina si diferite combinații ale acestora [Slots et al. 2004, Guentsch et al 2008, Griffiths et al. 2011, Flemmig et al. 2011, Herrera et al. 2008, Heitz-Mayfield et al. 2009]. Una dintre combinatiile cele mai des investigate a fost amoxicilina (AMX) si metronidazolul (MET). Aceasta combinatie reduce cantitatea bacteriilor parodontopatogene din regiunile afectate parodontal si duce la rezultate clinice pozitive din punctul de vederede al reducerii pungilor parodontale (PD) si al obtinerii de atasament gingival (CAL) [van Winkelhoff et al 1991, Winkel et al. 2001, Rooney et al. 2002, Matarazzo et al. 2008, Lopez et al. 1998, Guerrero et al. 2005].

Dozarea și perioada de administrare a antibioticelor descrise in literatura variază: 375 mg AMX and 250/500 mg MET 3x1/zi.pentru 7-8 zile [Cionca et al. 2010, 2009, Flemmig et al. 1998, Mombelli et al. 2005, Ehmke et al. 2005, Ribeiro et al. 2009]; alte studii au descris administrarea dozei de 500 mgAMX si MET 3x1/zi pentru 7 [Yek et al 2010, Griffiths et al 2011, Guerrero et a. 2005], sau a 500 mg AMX si 400/250 mg MET 3x1/zi pentru 10-14 zile [Heller et al. 2011, Silva et al. 2011, Matarazzo et al. 2008, Mestnik et al. 2010, Rodriguez et al. 2011]. Oricare ar fi doza si durata de administrare, este important ca acestea sa fie administrare in cantități suficiente.

Scopul studiului:

Scopul prezentului studiu este de a evalua rezultatele clinice, microbiologice si imunologice obtinute după tratamentul parodontal nechirurgical in combinație cu administrearea de amoxicilina (AMX) si metronidazol (MET) pe doua perioade diferite la pacienții cu parodontită cronică severă.

Obiective:

Primare:

- evaluarea schimbărilor nivelului de atasament gingival (CAL) după tratamentul parodontal nechirurgical (SRP in 24 h) in combinație cu administrearea de amoxicilina (AMX) si metronidazol (MET) pe două perioade diferite la pacienți cu parodontită cronică severă la 3, 6 si 12 luni.

Secundare:

- Evaluarea schimbărilor la nivelul PPD si a sângerării la sondare (BOP)

- evaluarea calitativă si cantitativă a efectului asupra florei bacteriene subgingivale

- determinarea efectului asupra citochinelor din fluidul intracrevilcular (IL-1β, IL-10, IL-8, MMP-8)

- morbiditatea postoperativă și rezultatele din punctului de vedere al pacientului

- reactii adverse

Pacienţi:

102 pacienţi cu parodontita cronică severă (Cel putin o PPD de 6 mm in fiecare cadran) vor fi recrutati la Clinica de Protetica a UMF Cluj-Napoca

Criterii de includere:

- virsta minima de 18 ani

- ≥12 dinți natural existenți

- semne clinice si radiografice a unei forme severe de parodontita cronica (PPD≥6 mm)

- nivel bun de igienă orala (indice de placa <25%)

- anamnestic sa fie pacienţi sănătoşi: fara boli de hipocoagulabilitate, diabet tip I sau II instabil, post iradiere in zona cap gat, care să necesite profilaxie antibiotica, boli infectioase sau boli cardiace care necesită profilaxie antibiotică inaintea tratamentelor dentare, sau medicatie cu efect asupra gingiei, afecțiuni hepatice

- consimţamântul informat al pacientului

Criterii de excludere:

- tratament parodontal nechirurgical in ultimele 12 luni

- administrare topica sau sistemica de antibiotic in ultimele 3 luni

- medicație care poate interactiona cu AMX sau MET (derivate de cumarina, care contin derivati alcoolici, 5-fluar uracil, disulfiram, emprenavir, lopinavir, ritonavir)

- medicație cu efect asupra parodontiului: ciclosporina A, fenitoina, blocanti de canal de calciu (Nifedipine, Verapamile, Amlodipine, Diltiazeme).

- paciente insarcinate sau aflate in perioada de lactaţie

- pacienti care nu indeplinesc criteriile de includere

Tipul studiului:

studiu clinic prospectiv, randomizat, controlat

Protocolul studiului:

Examinarea clinica va fi realizata de un investigator calibrat si blindat la tipul de tratament. Următorii parametrii se vor determina la baseline (inainte de SRP), la 3, 6 şi 12 luni după terapie:

- anamneza

- Anamneza de fumator

- statusul dentar si parodontal (PPD; CAL, furcatii, mobilitate)

- sângerarea şi supuraţia la sondare (BOP si SUP)

- Indicele de placa dentară şi inflamaţie gingivală (O´Leary 1972, Ainamo & Bay 1975)

- radiografie panoramica inaintea tratamentului

- radiografii retroalveolare de la dinţii test

- probe microbiologce si imunologice obţinute de la punga parodontala cea mai adâncă a fiecărei hemiarcade şi de la fiecare dinte test; Prin metoda real-time PCR se vor determina bacteriile parodontopatogene *Aggregatibacter actinomycetemcomitans, Porphyromonas gingivalis, Tannerella forsythia si Treponema denticola.* Prin testul ELISA se determina citochinele IL-1β, IL-10, IL-8, MMP-8.

Tratamentul parodontal:

Conform unei liste de randomizare, pacienţii vor fi împărţiţi in trei grupuri: grup martor si grup test1 si test 2. Împarţirea pacienţilor în cele trei grupuri va fi realizată conform unei liste de randomizare generata computerizat. Pentru toate grupurile se va efectua sub anestezie locala detartrajul si surfasajul radicular subgingival (SRP) in 24 de ore. Conform listei de randomizare pacienții vor primi urmatarea medicatie:

Grupul test 1: 500 mg AMX (3 x 1/zi) si 500 mg MET (3 x 1 /zi) pentru 7 zile

Grupul test 2: 500 mg AMX (3 x 1/zi) si 500 mg MET (3 x 1 /zi) pentru 3 zile

Grupul martor: fara antibiotic

Pacienţii vor fi chemaţi la control la 2 săptămâni, cand se vor lua primele probe imunologice din fluidul intracrevicular. Urmatoarele controale vor fi la 3, 6 si 12 luni.

Statistică:

Analiza statistică va fi realizată folosind un program computerizat SPSS pentru Windows. Valorile medii si devierile standard vor fi calculate pentru fiecare variabila clinica. De asemenea medianul va fi determinat. Teste neparametrice vor fi folosite.

Datele pacienţilor vor fi documentate pe formulare speciale, depozitate în siguranţa de coordonatorul studiului.

Aspecte etice:

Studiul va fi realizat în conformitate cu prevederile Declaraţiei de la Helsinki 1975, revizuita in 2000.

Toţi participanţii vor semna consimţământul informat al pacientului înainte de începerea studiului.

Recrutarea pacienţilor nu va fi începuta înainte de votul pozitiv al comisiei de etica a UMF Cluj-Napoca.

Cluj-Napoca 21.11.2011

Şef lucr. Dr. Raluca Cosgarea

Literatură

1. Ainamo, J., Bay, I. (1975) Problems and proposals for recording gingivitis and plaque. Int Dent J 25, 229-235.
2. Badersten A, Nilve´us R, Egelberg J. Effect of nonsurgical periodontal therapy (VIII). Probing attachment changes related to clinical characteristics. J ClinPeriodontol 1987: 14: 425–432.
3. Caffesse RG, Sweeney PL, Smith BA. Scaling and root planing with and without periodontal flap surgery. J ClinPeriodontol 1986: 13: 205–210.
4. Cionca N, Giannopoulou C, Ugolotti G, Mombelli A. Amoxicillin and metronidazole as an adjunct to full-mouth scaling and root planing of chronic periodontitis. J Periodontol 2009: 80: 364–371.
5. Cionca N, Giannopoulou C, Ugolotti G, Mombelli A. Microbiological testing and outcomes of full-mouth scaling and root planing with or without amoxicillin ⁄ metronidazole in chronic periodontitis. J Periodontol 2010: 81: 15–23.
6. Dahlen G, Wikström M, Renvert S. Treatment of periodontal disease based on microbiological diagnosis. A 5–year follow-up on individual patterns. J Periodontol 1996: 67: 879–887.
7. Ehmke, B., Moter, A., Beikler, T., Milian, E. &Flemmig, T. F. Adjunctive antimicrobial therapy of periodontitis: long-termeffects on disease progression and oral colonization. Journal of Periodontology 2005: 76, 749–759.
8. Engebretson, S. P., Grbic, J. T., Singer, R. &Lamster, I. B. GCF IL-1beta profiles in periodontal disease. Journal of Clinical Periodontology 2002: 29, 48–53.
9. Flemmig TF, Petersilka G, Völp A, Gravemeier M, Yillz M, Mross D, Prior K, Zamamoto J, Beikler T. Efficacz and safety of adjunctive local moxifloxacin delivery in the treatment of periodontitis. JPeriodontol 2011: 82: 96-105.
10. Goutoudi, P., Diza, E. &Arvanitidou, M. Effect of periodontal therapy on crevicular fluid interleukin-1beta and interleukin-10 levels inchronic periodontitis. Journal of Dentistry 2004: 32, 511–520.
11. Griffiths GS, Ayob R, Guerrero A, Nibali L, Suvan J, Moles DR, Tonetti MS. Amoxicillin and metronidazole as an adjunctive treatment in generalized aggressive periodontitis at initial therapz or re-treatment: a randomized controlled clinical trial. J ClinPeriodontol 2011: 38: 43-49.

12. Grossi SG, Zambon JJ, Ho AW, Koch G, Dunford RG, Machtei EE, Norderyd OM, Genco RJ. Assessment of riskfor periodontal disease. I. Risk indicators for attachment loss. J Periodontol 1994: 65: 260–267.

13. Guentsch A, Jentsch H, Pfister W, Hoffmann T, Eick S. Moxifloxacin as anadjunctive antibiotic in the treatment of severe chronic periodontitis. JPeriodontol. 2008: 79:1894-903.

14. Guerrero A, Griffiths GS, Nibali L, Suvan J, Moles DR, Laurell L, Tonetti MS. Adjunctive benefits of systemic amoxicillin and metronidazole in non-surgical treatment of generalized aggressive periodontitis: a randomized placebo-controlled clinical trial. J ClinPeriodontol 2005: 32: 1096–1107.

15. Haffajee AD, Socransky SS. Microbial etiological agents of destructive periodontal diseases. Periodontol 2000 1994: 5: 78–111.

16. Hamp, S.E., Nymann, S., Lindhe, J. Periodontal treatment of multirooted teeth. Results after 5 years. J ClinPeriodontol1975: 2, 126-135.

17. Heitz-Mayfield LJ, Trombelli L, Heitz F, Needleman I, Moles D. A systematic review of the effect of surgical debridement vs non-surgical debridement for the treatment of chronic periodontitis. J ClinPeriodontol 2002: 29(Suppl. 3): 92–102.

18. Heitz-Mayfield LJ. Systemic antibiotics in periodontal therapy. Austr Dent J 2009: 54 (Suppl 1): S96-S101.

19. Heller D, Varela VM, e Silva-Senem MX, Torres MCB, Feres-Filho EJ, Colombo APV: Impact of systemic antimicrobials combined to anti-infective mechanica debridement on the microbiota of generalized aggressive periodontitis: a 6-month RCT. J ClinPeriodontol 2011: 38: 355-364.

20. Herrera D, Alonso B, Leon R, Roldan S, Sanz M. Antimicrobial therapy in periodontitis: the use of systemic antimicrobials against the subgingival biofilm. J ClinPeriodontol 2008: 35 (Suppl 8): 45-66.

21. Lopez N, Gamonal J. Effects of metronidazole plus amoxicillin in progressive untreated adult periodontitis: results of a single one-week course after 2 and 4 months. J Periodontol 1998: 69: 1291–1298.

22. Marsh PD. Dental plaque: biological significance of a biofilm and community life-style. J ClinPeriodontol 2005;32(Suppl 6):7–15.

23. Matarazzo F, Figueiredo LC, Cruz SEB, Faveri M, Feres M. Clinical and microbiological benefits of systemic metronidazole and amoxicillin in the

treatment of smokers with chronic pperiodontiti: a randomized placebo-controlled studz. J ClinPeriodontol: 35: 885-896.

24. Mestnik MJ, Feres M, Figueriredo LC, Duarte PM, Lira EAG, Faveri M. Short-term benefits of the adjunctive use of metronidazole plus amoxixillin in the microbial profile and in clinical parameters of subjects with generalized aggressive periodontitis. J Clin periodontal 2010: 37: 353-365.

25. Mombelli A, Gmu¨ r R, Gobbi C, Lang NP. Actinobacillusactinomycetemcomitans in adult periodontitis. I. Topographic distribution before and after treatment. J Periodontol 1994: 65: 820–826.

26. Mombelli A, Schmid B, Rutar A, Lang NP. Persistence patterns of Porphyromonasgingivalis, Prevotellaintermedia⁄ nigrescens, and Actinobacillusactinomycetemcomitans after mechanical therapy of periodontal disease. J Periodontol 2000: 71: 14–21.

27. Mombelli, A. Antimicrobial profiles of periodontal pathogens and systemic antimicrobial therapy. Journal of Clinical Periodontology 2005: 32, 891–892.

28. O'Leary, T.J., Drake, R.B., Naylor, J.E. The plaque control record. J Periodontol1972: 43, 38.

29. Oliveira APL, Faveri M, Gursky L, Mestnik MJ, Feres M, Haffajee AD, Socransky SS, Teles RP. Effects of periodontal therapy on GCF cytokines in generalized aggressive periodontitis. J ClinPeriodontolepub ahead of print. doi: 10.1111/j.1600-051X.2011.01817.x.

30. Renvert S, Wikstro¨m M, Dahle´n G, Slots J, Egelberg J. Effect of root debridement on the elimination of Actinobacillusactinomycetemcomitans and Bacteroidesgingivalis from periodontal pockets. J ClinPeriodontol 1990: 17: 345–350.

31. Rooney J, Wade WG, Sprague SV, Newcombe RG, Addy M. Adjunctive effects to non-surgical therapy of systemic metronidazole and amoxycillin alone and combined. A placebo controlled study. J ClinPeriodontol 2002: 29: 342– 350.

32. Rosalem, W., Rescala, B., Teles, R. P., Fischer, R. G., Gustafsson, A. &Figueredo, C. Effect of non-surgical treatment on chronic and aggressive periodontitis: clinical, immunological and microbiological findings. Journal of Periodontology 2011: 82, 979–989.

33. Ribeiro Del Peloso E, Bittencourt S, Zanin IC, BoviAmbrosano GM, Sallum EA, Nociti FH, Goncalves RB, Casati MZ. Full-mouth ultrasonic debridement

associated with amoxicillin and metronidazole in the treatment of severechronic periodontitis. J Periodontol 2009: 80: 1254–1264.

34. Rodrigues AS, Lourencao DS, Neto LGL, Pannuti CM, Hirata RDC, Hirata MH, Lotufo RFM, Micheli G. Clinical and microbiological evaluation, by real-time PCR, of non-surgical treatment of aggressive periodontitis associated with amoxicillin and metronidazole. J Periodontol 2011, epubeahead of print DOI: 10.1902/jop.2011.110333.

35. Silva MP, Feres M, Sirotto TAO, Soares GMS, Mendes JAV, Faveri M, Figueiredo LC. Clinical and microbiological benefits of metronidazole alone or with amoxicillin as adjuncts in the treatment of chronic periodontitis: a randomized placebo-controlled clinical trial. J ClinPeriodontol 2011: 38: 828-837.

36. Slots J, Greenwell H, Fiorellini J, Giannobile W, Offenbacher S, Salkin L. Systemic antibiotics in periodontics. J Periodontol 2004: 75: 1553–1565.

37. van der Weijden GA, Timmerman FA. A systematic review on the clinical efficacy of subgingival debridement in the treatment of chronic periodontitis. J ClinPeriodontol 2002: 29(Suppl. 3): 55–71.

38. vanWinkelhoff AJ, Abbas F, Pavicic MJAMP, de Graaff J. Chronic conjunctivitis caused by oral anaerobes and effectively treated with systemic metronidazole plus amoxicillin. J ClinMicrobiol 1991: 29: 723–725.

39. Varela VM, Heller D, Silva-Senem MX, Torres MCMB; Colombo APVC, Feres-Filho EJ. Systemic antimicrobials adjunctive to a repeated mechanical and antiseptic therapz for aggressive periodontitis: a 6-month randomized controlled trial. J Periodontol 2011: 82: 1121-1130.

40. Winkel EG, van Winkelhoff AJ, Timmerman MF, van der Velden U, Van der Weijden GA. Amoxicillin plus metronidazole in the treatment of adult periodontitis patients. A double-blind placebo-controlled study. J ClinPeriodontol 2001: 28: 296–305.

41. Yek EC, Cintan S, Topcuoglu N, Kulekci G, Issever H, Kantarci A. Efficacy of amoxicillin and metronidazole combination for the management of generalized aggressive periodontitits. J Periodontol 2010: 81: 964-974.
